# DMRG-Tailored Coupled Cluster Method in the 4c-Relativistic Domain: General Implementation and Application to the NUHFI and NUF_3_ Molecules

**DOI:** 10.1021/acs.jctc.4c00641

**Published:** 2024-10-09

**Authors:** Jakub Višňák, Jan Brandejs, Mihály Máté, Lucas Visscher, Örs Legeza, Jiří Pittner

**Affiliations:** † J. Heyrovský Institute of Physical Chemistry, Academy of Sciences of the Czech Republic, v.v.i., Dolejškova 3, 18223 Prague 8, Czech Republic; ‡ Faculty of Mathematics and Physics, Charles University, Ke Karlovu 3, 12116 Prague, Czech Republic; § Middle East Technical University, Üniversiteler Mahallesi, Dumlupınar Bulvarı No:1, 06800 Çankaya Ankara, Türkiye; ∥ Faculty of Science, Humanities, and Education, Technical University of Liberec, Studentská 1402/2, 461 17 Liberec, Czech Republic; ⊥ Strongly Correlated Systems “Lendület” Research Group, Wigner Research Centre for Physics, Konkoly-Thege Miklós út 29-33, H-1121 Budapest, Hungary; # Department of Mathematics, Technical University of Munich, Boltzmannstr. 3, 85748 Garching, Germany; ∇ Department of Chemistry and Pharmaceutical Sciences, De Boelelaan 1108, Vrije Universiteit Amsterdam, NL-1081 HZ Amsterdam, Netherlands; ○ Institute for Advanced Study, Technical University of Munich, Lichtenbergstrasse 2a, 85748 Garching, Germany

## Abstract

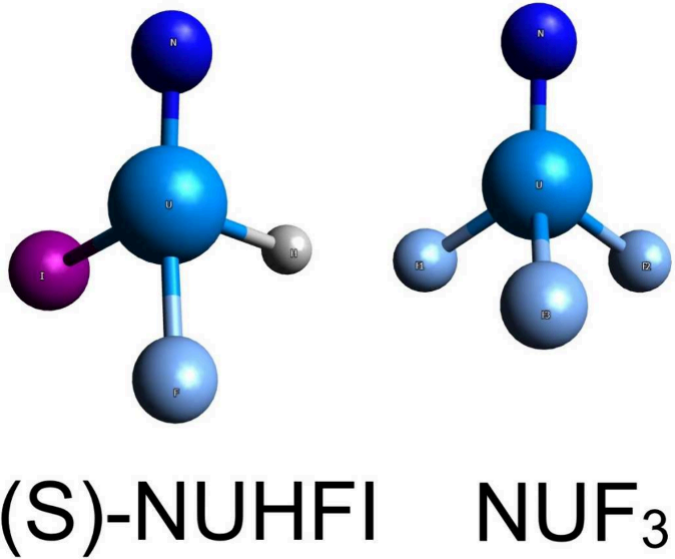

Heavy atom compounds represent a challenge for computational chemistry due to the need for simultaneous treatment of relativistic and correlation effects. Often such systems also exhibit strong correlation, which hampers the application of perturbation theory or single-reference coupled cluster (CC) methods. As a viable alternative, we have proposed externally correcting the CC method using the density matrix renormalization group (DMRG) wave functions, yielding the DMRG-tailored CC method. In a previous paper [*J. Chem. Phys.***2020**, *152*, 174107], we reported a first implementation of this method in the relativistic context, which was restricted to molecules with real double group symmetry. In this work, we present a fully general implementation of the method, covering complex and quaternion double groups as well. The 4c-TCC method thus becomes applicable to polyatomic molecules, including heavy atoms. For the assessment of the method, we performed calculations of the chiral uranium compound NUHFI, which was previously studied in the context of the enhancement of parity violation effects. In particular, we performed calculations of a cut of the potential energy surface of this molecule along the stretching of the N–U bond, where the system exhibits strong multireference character. Since there are no experimental data for NUHFI, we have performed also an analogous study of the (more symmetric) NUF_3_ molecule, where the vibrational frequency of the N–U bond can be compared with spectroscopic data.

## I Introduction

The density matrix renormalization group method (DMRG)^1^ was introduced to the quantum-chemical community^2−4^ about two decades ago. Since then, it has seen many applications to multireference systems, and the DMRG-SCF method is becoming a standard computational approach, replacing traditional CASSCF. The biggest advantage of the DMRG method is its capability to treat large active spaces; current implementations can go beyond 50 active space spinors.^5,6^ However, a major drawback of DMRG is its inability to capture dynamical correlation since it cannot include all virtual spinors. The dynamic correlation plays an important role in virtually all molecules, and its accurate description is important when trying to achieve chemical accuracy. Although the DMRG method is already well established, the methods for treating the dynamical correlations on top of DMRG are still in the pioneering stage. Past efforts were either based on second order perturbation theory,^7^ internally contracted MRCI (multireference configuration interaction),^8^ random phase approximation,^9^ the canonical transformation method,^10^ the perturbation theory with matrix product states,^11^ the adiabatic connection method,^12^ or the restricted active space density matrix renormalization group method.^13,14^

In our group, we employed an approach based on the coupled cluster method externally corrected by DMRG.^15^ As the name suggests, this is a combination of DMRG and the coupled cluster (CC) method. The CC method is well-known for its efficiency in describing the dynamical correlation. In the externally corrected approach, first a DMRG calculation is performed within the strongly correlated active space fixed, which accounts for the static correlation. The second step is the CC analysis of the matrix product state (MPS) wave function, obtained from DMRG, which yields CC amplitudes with indices restricted to the active space spin orbitals. Then, a CC calculation is performed on the whole orbital space, keeping the active space amplitudes fixed, which captures the dynamical correlation. Already the simplest version thereof, the tailored CCSD (CC with single and double excitations) approach,^16,17^ yields very promising results.^15,18−21^ In the nonrelativistic context, it has been implemented in the ORCA program,^22^ employing the domain-based local pair natural orbital (DLPNO) techniques to achieve high computational efficiency.^23−25^

In a previous paper,^26^ we presented the DMRG-tailored coupled cluster method in the 4c-relativistic domain in an implementation restricted to real double groups, i.e., to diatomics or other highly symmetric molecules. Here, we report a general implementation applicable to polyatomic molecules with complex or quaternion double group symmetry.

Computational studies of polyatomic molecules with low symmetry at the four-component relativistic and post-HF correlated levels are relatively scarce in the literature. To illustrate the applicability of our approach, we have selected the NUHFI molecule, which is a chiral compound and was previously studied at the relativistic X2C-DFT level in the context of the enhancement of parity violation effects.^27^ For the search for parity violation effects in high-resolution spectroscopy experiments, it is convenient if the molecule has a vibrational frequency in the range of the CO_2_ laser (900–1100 cm^–1^), which corresponds to the stretch of the U≡N triple bond in this molecule. On the other hand, stretching a triple bond beyond the immediate vicinity of equilibrium quicky introduces appreciable nondynamic correlation, and the N_2_ molecule has been often employed as a benchmark to test the performance of multireference correlated methods^20,28−35^ and to demonstrate the failure of single-reference ones to properly describe dissociation of this bond.^36^ This has motivated us to choose the NUHFI molecule as a relativistic variant of such a benchmark and to study the cut of the potential energy surface of this molecule along the stretch of the N≡U bond (similar studies using pCCD and pCCD-tailored CC have been done by Leszczyk et al.^37^). Since there are no experimental data available for this molecule, we have also performed a study of the NUF_3_ molecule, which has an analogous N–U bond and where vibrational frequency and anharmonicity have been measured. However, due to its higher symmetry, this molecule does not fully exploit our most general implementation of the 4c-TCCSD method.

## II Theory and Implementation

The state-of-the-art post-HF relativistic calculations employ the no-virtual-pair approximation and the empty Dirac picture, i.e., the negative-energy solutions of the Dirac–Coulomb Hamiltonian are projected out, and only the electronic solutions enter the correlated calculation. No excitations to the positronic manifold thus occur, corresponding to the neglect of virtual electron–positron pair creation. This is a well substantiated approximation at the chemical energy scale, and it yields a second-quantized Hamiltonian formally analogous to the nonrelativistic case
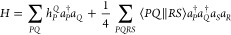
1where the indices *P*, *Q*, *R*, and *S* run over the positive-energy four-component spinors spanning the one-electron basis and ⟨*PQ*∥*RS*⟩ = ⟨*PQ*|*RS*⟩ – ⟨*PQ*|*SR*⟩ stands for the antisymmetrized two-electron integral in Physicist’s notation. In the Kramers-restricted formalism, barred spinors (ϕ_*p̅*_) and unbarred spinors (ϕ_*p*_) form Kramers pairs related to each other by action of the time-reversal operator *K*:
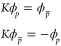
2The Kramers symmetry, which leads to a 2-fold degeneracy of spinor energies in the absence of magnetic fields,^38^ can be used to play the role that spin symmetry has in nonrelativistic theory. In such approaches, the *M*_*S*_ good quantum number is replaced by the *M*_*K*_ quasi quantum number,^39^ which is 1/2 for unbarred spinors (A) and −1/2 for spinors with barred indices (B). The capital indices in eq 1 run over both A and B components of Kramers pairs. In contrast to the nonrelativistic case, the Hamiltonian (eq 1) is in general not block-diagonal in *M*_*K*_, but this number can then serve to partition the operators and Hamiltonian blocks in much the same way as is done with the *M*_*S*_ quantum number. Since each creation or annihilation operator in eq 1 changes *M*_*K*_ by ±1/2, the Hamiltonian can only directly couple states with |Δ*M*_*K*_| ≤ 2. States with a still higher difference in |*M*_*K*_| are only coupled indirectly and can possibly be neglected if the Kramers’ pairing is chosen such that it minimizes the couplings between states with different *M*_*K*_ values. Also with the aid of Kramers’ symmetry, the index permutation symmetry of the 2e integrals in eq 1 is still lower than in the nonrelativistic case as this is based on the use of real-valued spin–orbitals, whereas relativistic spinors are in general complex-valued.

The DIRAC program^40,41^ employs a quaternion symmetry approach which combines the Kramers and binary double group symmetry (*D*_2h_^*^ and subgroups).^42^ The binary double groups can be divided into three classes based on the Frobenius–Schur indicator: “real groups” (*D*_2h_^*^, *D*_2_^*^, and *C*_2v_^*^); “complex groups” (*C*_2h_^*^, *C*_2_^*^, and *C*_s_^*^); and “quaternion groups” (*C*_i_^*^ and *C*_1_^*^).^43^ Generalization of nonrelativistic post-HF methods is simplest for the “real groups”, where the integrals in eq 1 are real-valued and the ones with an odd number of barred (B) indices vanish; i.e., one has only to include additional “spin cases” of integrals ⟨AA|BB⟩ and ⟨AB|BA⟩ (in Physicist’s notation) as well as take care of the fact that in general integrals ⟨BA|BA⟩ ≠ ⟨AA|AA⟩. For the complex groups, the integrals are complex-valued, but still only integrals with an even number of barred indices are nonzero. Finally, in the lowest symmetry case of “quaternion groups,” all the integrals have to be included and are complex-valued.^43−46^

The tailored coupled cluster (CC) method belongs to the broader category of externally corrected CC methods, which take information on static correlation from some non-CC external source and include it into the subsequent CC treatment.^47^ The tailored CC method (TCC), proposed by Bartlett et al.,^16,48−50^ is conceptually the simplest such method. It employs the split-amplitude ansatz for the wave function introduced by Piecuch et al.^51,52^
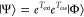
3where |Φ⟩ is the Dirac–Hartree–Fock–Slater determinant and *T*_cas_ containing amplitudes with all active indices is “frozen” at values obtained from CASCI or in our case from DMRG. The external cluster operator *T*_ext_ is composed of amplitudes with at least one index outside the DMRG active space.

The simplest version of the method truncates both *T*_cas_ and *T*_ext_ to single and double excitations. The excitation operators *T*_ext_ and *T*_cas_ commute, which allows the use of the standard CCSD solver, modified to keep the amplitudes from *T*_cas_ fixed at values obtained from the DMRG MPS wave function.^53^ Since the Hamiltonian contains only one- and two-body terms, the TCCSD energy with *T*_ext_ = 0 and *T*_cas_ from DMRG reproduces the DMRG energy. In the limit of the DMRG active space including all MOs, TCC thus recovers the FCI energy. In general, a quadratic error bound valid for TNS-TCC methods has been derived.^19^

In refs (15) and (18), we have presented an implementation of the TCCSD method for the nonrelativistic case, which was later followed by more efficient LPNO and DLPNO based implementations,^23,24^ and the implementation of TCCSD for the simpler real-group symmetry case followed.^26^ In contrast to the nonrelativistic DMRG, where the “sites” correspond to spatial orbitals with a four-dimensional site basis, in the relativistic case^54^ a site represents a single Kramers spinor, being occupied or unoccupied. The number of sites for the DMRG sweeps thus doubles with respect to the nonrelativistic case. In general, it is also possible to form a four-dimensional “super site” by taking the tensor product basis of the barred and unbarred Kramers spinors, which might look more beneficial by reducing the number of sites by two. However, in this implementation, the quantum number decomposed site operators^5,55^ do not reduce to scalar multiplicative factors via the diagonalization procedure, and consequently the corresponding general tensor algebra turns out to be computationally more demanding. Extraction of the CI coefficients from the MPS wave function also requires an optimal implementation for system sizes in the range of 100 sites or more. As there is a large redundancy in occupation number “strings”, i.e., arrays with binary elements corresponding to the various determinants, we form several precontracted components for the left and right DMRG subsystem MPS components; thus, the final contraction of the network can be accelerated tremendously. In addition, these processes can be performed very efficiently via massive parallelization. Our code can also handle arbitrary Abelian and non-Abelian symmetries, which again could reduce computational time and memory requirement significantly.

From the technical point of view, in our previous works, outlined above, the Budapest DMRG code has been interfaced with the DIRAC code, allowing us to perform the TCC workflow for heavy diatomic molecules when relativistic treatment became mandatory.^26^ In the case of the quaternion symmetry, however, the interactions are complex valued; thus, the underlying tensor algebra of the DMRG had to be developed for complex numbers as well. This affected functions related to partial summations to generate the auxiliary operators, the renormalization of the block operators, tasks based on the wave function, and the diagonalization of the effective Hamiltonian. For the complex DMRG version with long-range interactions, we also found that the stability of the DMRG performance was more sensitive to accumulated numerical noise; thus, numerical roundings have been enforced at several points in the DMRG algorithm. For the diagonalization step, we further developed the complex version of the Jacobi–Davidson variant with preconditioning, which was found to be very sensitive to even very small numerical noise on the order of 10^–12^. Rounding has been introduced at various points of the algorithm, and finally, we achieved a stable version which is more efficient than the Lánczos once a good starting vector together with preconditioning is provided. Alternatively, the Lánczos algorithm can be used which requires more iteration steps in the course of the diagonalization but provides very stable convergence even in the case of small numerical noise in the real and imaginary components. As a hybrid solution, we use the Lánczos algorithm during the warmup procedure via the dynamic extended active space procedure (DEAS)^53^ and the Davidson method for all further DMRG sweeps once initial starting vectors for the diagonalization step is available by the DMRG wave function transformation protocol.^5^ Similar developments have been done on the post-DMRG utility functions, i.e., calculation of single orbital entropy, mutual information, and obtaining the T1 and T2 amplitudes. The full workflow of the general 4c-TCC in the case of quaternion symmetry has been tested and benchmarked against full-CI reference data on small systems. For larger system sizes, however, the methods for optimization of site ordering turned out to be a delicate issue and the procedure from the nonrelativistic DMRG turned out in general not to be transferable. Therefore, particular care has to be taken to the ordering of the sites, which has to keep the A and B spinors from each Kramers pair adjacent. We used thus the order of adjacent pairs of A and B spinors sorted by HF orbital energy, which is probably not optimal, but the optimization was not able to improve it. Besides the complex-valued arithmetics and larger number of integrals, this also contributed to the high computational cost of the 4c-DMRG calculations.

Once the CI coefficients *c*_*i*_^*a*^ and *c*_*ij*_^*ab*^ have been extracted from the MPS wave function, the standard CC analysis is performed to convert them to the CC amplitudes
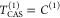
4
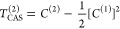
5In the present relativistic CC implementation in the DIRAC code, one does not distinguish “spin cases” of the excitation amplitudes, but amplitude indices run through the united range of A and B spinors, which transparently includes the excitations with nonzero Δ*M*_*K*_. To our advantage, this also matches the single-spinor site representation in the DMRG. In the lowest quaternion-symmetry case, the SD amplitudes are in general all nonzero and complex-valued, while in higher symmetry they become sparse, analogously to the one- and two-electron integrals. Due to the combined effect of doubling the amplitude index range compared to nonrelativistic spin-restricted CCSD and of complex arithmetics required, the low-symmetry relativistic calculations are computationally more demanding by a prefactor of almost 2 orders of magnitude, which restricts the applications to small molecules.

Here, we remark that the main purpose of the given work is to present a general implementation of the relativistic version of the TCC method to handle also complex integrals and quaternion symmetry. To demonstrate the underlying workflow and analyze stability and convergence issues, we limit the bond dimension of the DMRG to around 2000, truncate the CC excitation ranks to single and doubles, and set the residual error of the diagonalization step to 10^–8^. Our implementation is, however, a general one and can easily be extended toward more complex calculations with larger bond dimension and larger active spaces. In fact, from the DMRG viewpoint, recently we have introduced massively parallel hybrid CPU–GPU DMRG building on state of the art software and hardware technologies,^56−59^ reaching a quarter petaflops performance and large active spaces even on a single node via applied bond dimension values on the order of tens of thousands. Our implementation has a nearly ideal scaling with the number of computational units and can be extended to several nodes; thus, extension of our hybrid CPU-GPU tensor and matrix libraries to the complex case will boost tremendously the efficiency of 4c-DMRG-TCC in the near future. From the point of view of the TCC, the initial implementation of 4c-CCSD in DIRAC represents a bottleneck, but recently a distributed-GPU implementation of the general-order CC has been reported by DIRAC developers,^60^ designed to be compatible with 4c calculations. Therefore, the 4c-TCC method should in the future be reimplemented using this machinery to be able to compute larger molecules. In addition, an efficient construction of amplitudes with higher excitation ranks, like t3, t4, etc., from the DMRG wave function has also been developed and applied already in standard quantum chemistry, which can again be transferred to the complex framework (unpublished yet). Therefore, such developments will be available for the relativistic framework, as well.

Regarding computational complexity and wall time, both DMRG and CC can be expensive; usually also, the memory demand is higher in TCC, but that also depends on the DMRG bond dimension. In general, the computational complexity and memory requirements of DMRG scale as  and , respectively. In the current work, we used an old version of our code apart from extensions to the complex case. The scaling of such calculations is similar to the nonrelativistic one *O*(*D*^3^*N*^4^) with just a prefactor on the order of two. This can, however, be accelerated tremendously via AI accelerators as reported in refs (57) and (59). Calculation of the amplitudes from the DMRG wave function using algorithmic solutions outlined in the previous paragraph takes only a fraction of time, usually on the order of only a few minutes even up to CC excitation rank 4. The CC part is performed via DIRAC with taking again a relatively smaller amount of time since the main bottlenecks were the memory requirements. We had to truncate the virtual space for CC to make the calculations tractable, and since CCSD scales as , this truncation shortens the CPU time for CC substantially.

## III Computational Details

**Figure 1:**
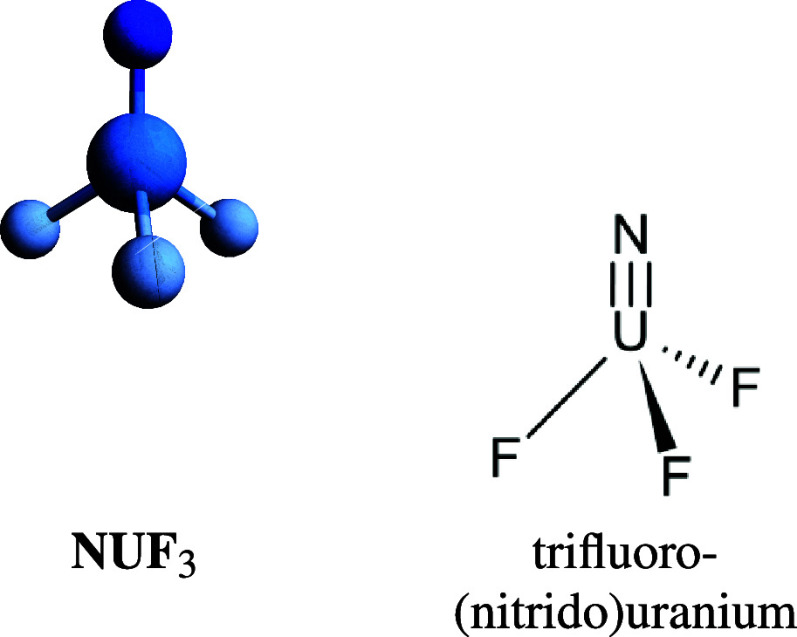
*C*_3*v*_ molecular geometry of NUF_3_ with N–U bond length changed from 1.753 Å to a reference value of 1.740 Å.

The *C*_3*v*_ molecular geometry of NUF_3_ (cf. Figure 1) has been adopted from a CASPT2 computation found in the literature.^61^

**Figure 2:**
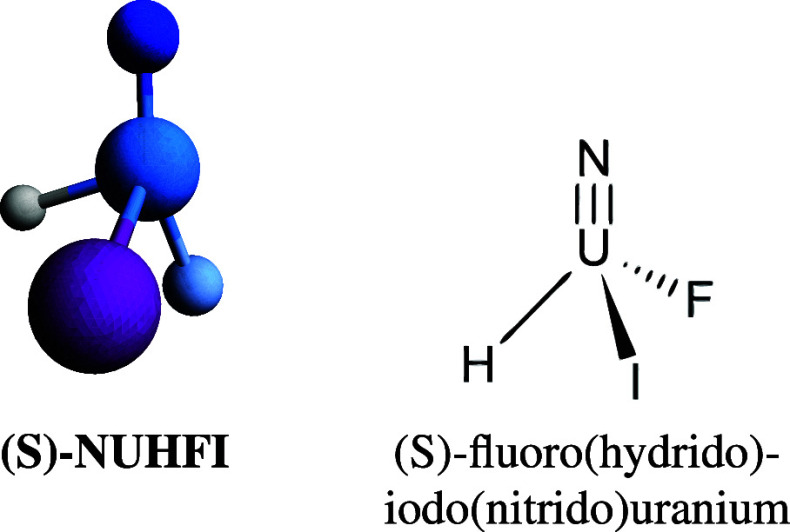
ECP/B3LYP+D3/def-TZVPP level optimized NUHFI geometry with N–U bond length changed from 1.709 Å to a reference value of 1.700 Å.

The molecular geometry of NUHFI (cf. Figure 2) has been optimized at the ECP/B3LYP+D3/def-TZVPP level using Turbomole.^62^

The positions of U, H, F, and I atoms have then been fixed, and the N atom was moved either closer or more apart from the uranium central atom along the optimized direction of the N–U bond. The computations along the N–U stretching mode were performed in the four-component relativistic all-electron approach with a Dirac–Coulomb Hamiltonian^43^ using our development version of the DIRAC and Budapest DMRG programs. Dyall’s double-ζ atomic basis sets^63^ have been used for U and I and nonrelativistic contracted cc-pVDZ^64^ for N, H, and F (this combination is called “DZ” henceforth).

The selection of the DMRG active space has been based on the single-orbital entropy spectrum calculations in a larger (32,98) space with a bond dimension^5^ up to *M* = 512 for *R*_NU_ = 1.4, 1.7, 2.2, and 4.8 Å. The notation (*n*,*m*) denotes *n* correlated electrons in *m* Kramers pairs, while other electrons are kept inactive. The amplitude space for both CCSD and TCCSD (47th to 252nd Kramers pair) has been chosen based on the trade-off between computational demands and spectroscopic properties prediction accuracy taking into account energy gaps in the molecular bispinor set.

For each method considered, the UN bond length and force constant were determined by a fit of the UN stretching mode with the programs VIBANAL^65^ and TWOFIT associated with DIRAC software. Harmonic frequencies were obtained by using this computed force constant to correct the ECP/B3LYP+D3/def-TZVPP Hessian computed by Turbomole using the relations
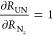
6
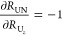
7
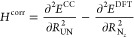
8
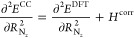
9
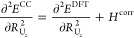
10
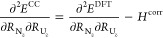
11and recomputing the frequencies by rediagonalizing the mass-weighted Hessian. In this mass-weighted Hessian, the most abundant isotope mass was selected for each atom. The first two relations are valid if the bond is aligned along the *z* axis, which is a trivial condition to fulfill. The other relations require the UN mode to be weakly coupled to the other modes. This assumption was verified by checking the normal modes computed at the DFT level of theory. For both molecules considered, the normal mode considered has less than 2% admixture of coordinates other than the *R*_N_*z*__ coordinate. The anharmonicities were computed using the program VIBANAL^65^ from a polynomial fit of the potential curves along the UN bond employing the effective mass
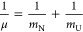


Regarding numerical settings and convergence issues, we note that, for integrals, the DIRAC default cutoffs have been applied (10^–12^). However, we checked that no-cutoff does not lead to significantly different results, while it more than doubles the computational time. The same is true for default approximation to the (SS|SS) type of integrals. The default approximation is described by the DIRAC manual as “.LVCORR”, which activates the Dirac–Coulomb Hamiltonian in which (SS|SS) integrals are neglected and replaced by an interatomic SS correction.^40,66^ Furthermore, the cutoff applied in the DMRG to the integrals was 10^–12^, and the residual error of the Davidson/Lánczos diagonalization procedure was set to 10^–8^. The energy changes in the last two DMRG sweeps were on the order of 10^–5^.

### III.A Preparation of the Molecular Spinors

**Figure 3:**
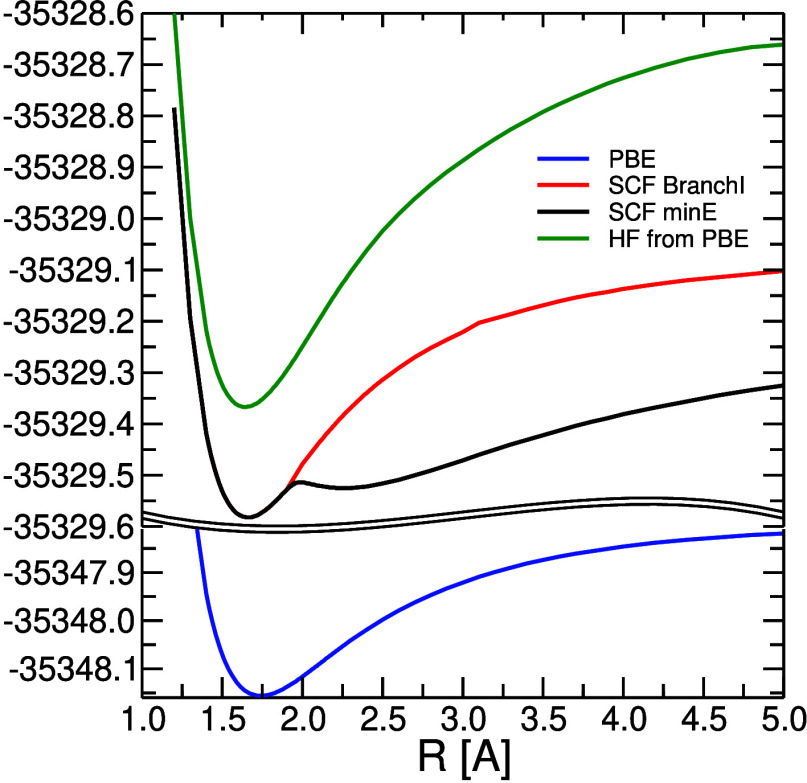
SCF and DFT (PBE) potential energy curves.

For the correlated calculations, one needs a one-electron basis of molecular spinors, and its choice might strongly influence the final results (for all methods except full CI). It turns out that the Dirac–Hartree–Fock (DHF) N–U potential energy curve bifurcates at a moderate stretch from equilibrium (*R* = 1.90 Å for NUHFI) into a lower branch (black line in Figure 3) with a double-well character and an upper branch with a shape typical for the ground state of diatomic molecules (red line). The DHF solution of each branch can be followed by restarting the SCF procedure from a converged solution at a neighboring geometry. Unfortunately, the molecular spinors from either branch lead to unphysical artifacts in the potential energy curves, when a subsequent DMRG calculation with a limited active space is performed. The best solution of this problem would probably be a 4c-CASSCF calculation; unfortunately, we did not find any working implementation of this method *for quaternion double group symmetry*, neither in DIRAC nor in the BAGEL and ChronusQ codes (in the version available to us). Fortunately, the DFT/PBE N–U potential energy curve (blue line in Figure 3) exhibits no such instability and bifurcation. Even the single-determinant Dirac–Hartree–Fock energy computed from the self-consistently converged PBE Kohn–Sham molecular spinors (green line in Figure 3) is free of bifurcation or double-well problems. However, the HOMO–LUMO gap is significantly narrowing after *R* > 2.0 Å in these orbitals, which leads to convergence problems of CC methods at larger N–U distances. There are additional reasons for selecting the PBE functional as a source of molecular spinors: We have tried several meta-GGA/hybrid/advanced functionals, and the Hartree–Fock energy potential energy curves computed from their converged spinors exhibited a nonphysical maximum at larger N–U distances (cf. Supporting Information, Figure S1). GGA functionals (including PBE) have been found to be free of this artifact, and from the ones we tested, the PBE spinors provided the lowest Hartree–Fock energy.

Finally, we found the PBE functional adequate to describe other molecules containing the U–N bond (see Appendix). We have thus based our post-HF calculations on the PBE Kohn–Sham molecular spinors.

## IV Results and Discussion

### IV.A NUF_3_ Molecule

A crucial test of any theoretical method is its comparison with experimental findings. Due to the lack of experimental spectroscopic data for NUHFI, we investigated first a similar molecule, NUF_3_, for which the vibrational frequency of the N–U stretch has been measured.^67^ The experimental anharmonicity is not available, so we tried to make a plausible estimate based on the measured anharmonicity 5.3 cm^–1^ in NU^+^^68^ with an error bar of ±8%, which corresponds to the spread of the N–U bond vibrational frequencies in the set of related species (UN^+^,^68^ UN,^69^ NUO^+^,^69^ NUN,^69^ NUF_3_,^61,69^ NUNH^69^)

The orbital range in 4c-CCSD, 4c-CCSD(T), and 4c-TCCSD calculations for NUF_3_ has always been 35–172 and will be henceforth omitted.

Table 1 presents the resulting spectroscopic constants of NUF_3_. Similarly to the NUHFI molecule, uncorrelated results from DHF-SCF and DHF from PBE spinors strongly underestimate the bond length and overestimate the vibrational frequency. The bifurcation of the DHF-SCF solution occurs at *R* = 1.93 Å, as can be seem from Figure S2 in the Supporting Information.

**Table 1:** Spectroscopic Parameters for the N–U Bond of the NUF_3_ Molecule in the Dyall-v2z(U) and cc-pVDZ (N,F) Basis

method	*r*_e_, Å	*E*_min_, a.u.	ω_e_, cm^–1^	ω_e_*x*_e_, cm^–1^	Δω_e_, cm^–1^	Δω_e_*x*_e_, cm^–1^
4c-DHF-SCF^e^	1.6704	–28412.17024	1282.86	5.64	344.86	0.34
4c-PBE	1.7567	–28428.26886	973.23	3.88	35.23	–1.42
4c-DHF(PBE)	1.6553	–28411.96614	1307.93	2.98	369.93	–2.32
4c-CCSD^a^	1.7212	–28413.59978	1106.06	3.06	168.06	–2.24
4c-CCSD(T)^a^	1.7787	–28413.70853	930.2	2.74	–7.80	–2.56
4c-DMRG(14,15)	1.7565	–28412.08719	805.61	3.25	–132.39	–2.05
4c-DMRG(26,26)^e^	1.7777	–28412.16795	821.37	6.89	–116.63	1.59
4c-DMRG(32,32)	1.7908	–28412.23037	910.19	8.81	–27.81	3.51
4c-(14,15)TCCSD^a^	1.7661	–28413.63386	923.95	2.83	–14.05	–2.47
4c-(20,32)TCCSD^a^	1.7576	–28413.64760	964.93	4.47	26.93	–0.83
**4c-(26,26)TCCSD**^a^	**1.7587**	**–28413.64416**	**954.6**	**5.36**	**16.60**	**0.06**
4c-(32,32)TCCSD^a^	1.7493	–28413.64722	1008.88	6.89	70.88	1.59
ECP^b^/CCSD(F12)	1.6995	–829.92403	1104.63	5.34	166.63	0.04
ECP^b^/CCSD(T)(F12)^d^	1.7218	–829.99853	1061.62	2.80	123.62	–2.50
ECP^b^/CCSD(T)(F12)^g^	1.7200	–829.99844	1058.26	4.95	120.26	–0.35
ECP^b^/CCSD(T*)(F12)^d^	1.7247	–830.00965	1083.8	5.07	145.80	–0.23
ECP^b^/CCSD(T*)(F12)^g^	1.7216	–830.00941	1053.39	5.59	115.39	0.29
ECP^b^/DFT:r2SCAN	1.7093	–831.52519	1000.59	3.43	62.59	–1.87
ECP^b^/DFT:mpsts-noa2	1.7279	–831.33322	1019.11	3.50	81.11	–1.80
CASPT2(6,16)^i^	1.753					
**exptl.**^j^			**938**			
estimate^k^				5.3 ± 0.4		

a All 4c-CC and 4c-TCC computations have been done with amplitude range 35–172.

b Scalar quasi-relativistic, with def2-TZVPP AO basis, computed via Turbomole V7.6.^62,70^

d The curve is limited by *R*_max_ = 2.00 Å.

e The curve is limited by *R*_max_ = 1.90 Å.

g The curve is limited only by *R*_max_ = 2.10 Å, but a higher branch of the bifurcated HF-SCF solution was taken for longer distances of *R* > 1.93 Å.

i From ref (61).

j Experimental data from ref (67) indicated in boldface.

k Estimate based on the experimental anharmonicity value for NU^+^^68^ and a spread of vibrational frequencies in a group of similar molecules.

The PBE functional, which has been employed to obtain molecular spinors for subsequent post-HF calculations due to its bifurcation-free behavior, yields a bond length in a good agreement with CASPT2(6,16) calculations^61^ and vibrational frequency reasonably close to the experimental value.^67^

Due to excessive memory demands we had to truncate the virtual orbital space for the CC calculations; the orbital range in 4c-CCSD, 4c-CCSD(T), and 4c-TCCSD calculations for NUF_3_ has been always 35–172 and will be henceforth be omitted. Concerning the single-reference CC methods, addition of perturbative triples substantially elongates the bond and lowers the vibrational frequency, exemplifying the importance of the dynamic correlation. DMRG consistently yields bond lengths close to the CCSD(T) value, while the vibrational frequencies are too small compared to the experimental value, except in the largest (32,32) space, where ω_e_ is close to the experiment. However, DMRG in this space yields a too long bond length and an anharmonicity that is too high of an anharmonicity.

DMRG yields widely varying anharmonicities for different active spaces. However, in contrast to NUHFI, here the DMRG in the smallest active space yields the smallest anharmonicity.

The 4c-TCCSD method yields similar bond lengths in all active spaces, which are slightly shorter than 4c-CCSD(T) value and close to the CASPT2(6,16) value from the literature.^61^ The boldface line indicates our best 4c-TCCSD result in the (26,26) space, in the sense of best match with the reference harmonic frequency and anharmonicity. The vibrational frequencies are in the range of 920–1010 cm^–1^, with the one for the optimal space (26,26) being 955 cm^–1^, which is reasonably close to the experimental value of 938 cm^–1^. The anharmonicities are in this case more sensitive to the active space choice than in the NUHFI molecule. Small active spaces yield values close to the single reference methods, while larger spaces yield larger anharmonicity values. For the optimal space (26,26), the result is 5.3 cm^–1^, which is in line with the estimate based on the experimental data of similar molecules.^68^

It is interesting to note that there is a considerable difference in the harmonic vibrational frequency between the 4c-CCSD and 4c-CCSD(T) results. This indicates that the dynamic correlation, better captured by CCSD(T), is important to describe the PEC shape in the vicinity of the equilibrium. On the other hand, the anharmonicities at 4c-CCSD and 4c-CCSD(T) levels are similar and considerably lower than the reference estimate. This might be plausibly explained by the lack of static (strong) correlation in the single-reference treatments. The strong correlation becomes more pronounced with the N–U bond stretch (cf. orbital entropies in the Supporting Information), and the anharmonicity is influenced by the PEC shape at the stretched bond length.

We also performed calculations employing scalar ECP quasirelativistic CC methods with explicit correlation and in a larger basis set. The ECP-HF-SCF solution has again a bifurcation at 1.93 Å and the post-HF results depend on which SCF branch has been chosen as reference (see footnotes in Table 1). All versions of the CCSD(T) method in this case yield somewhat shorter bond length than the CCSD(T) in the full four-component treatment, while the vibrational frequencies are consistently too high. The anharmonicity depends on whether the lower or upper branch of the bifurcated SCF solution has been employed. The upper branch, which does not have an unphysical maximum in the SCF energy, is probably a better choice and yields CCSD(T)-F12 anharmonicities around 5 cm^–1^, which is close to the estimate. The ECP/DFT results yield similar bond lengths as the CC methods, their vibrational frequencies being substantially over the experimental one and their anharmonicities below the estimate. It thus seems that a combined description of the dynamic and static correlation is needed for this molecule as well in order to describe its spectroscopic properties, particularly the fact that DMRG in modest spaces is not able to yield reliable anharmonicities.

### IV.B NUHFI Molecule

**Figure 4:**
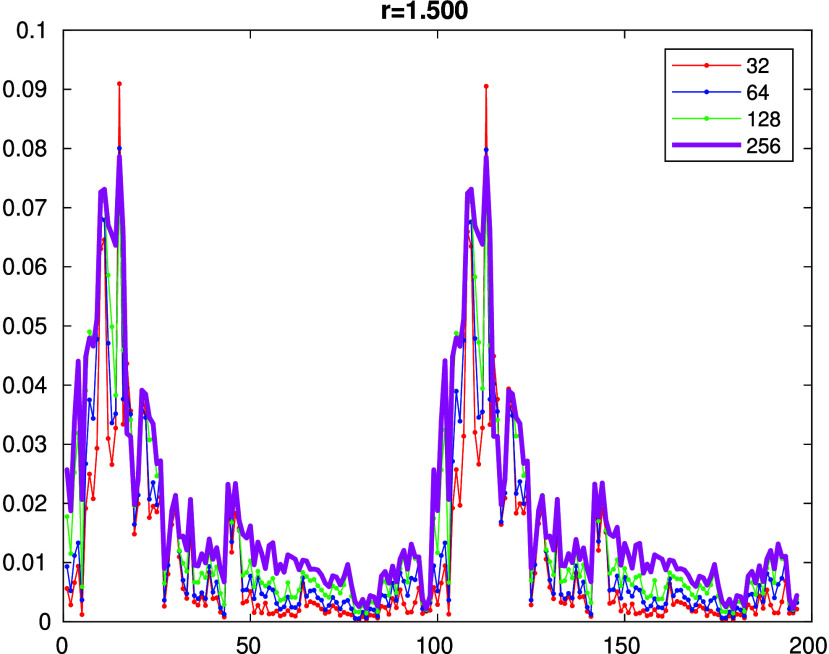
DMRG(32,98) entropy profiles at *r*_N–U_ = 1.5 Å for various bond dimensions.

**Figure 5:**
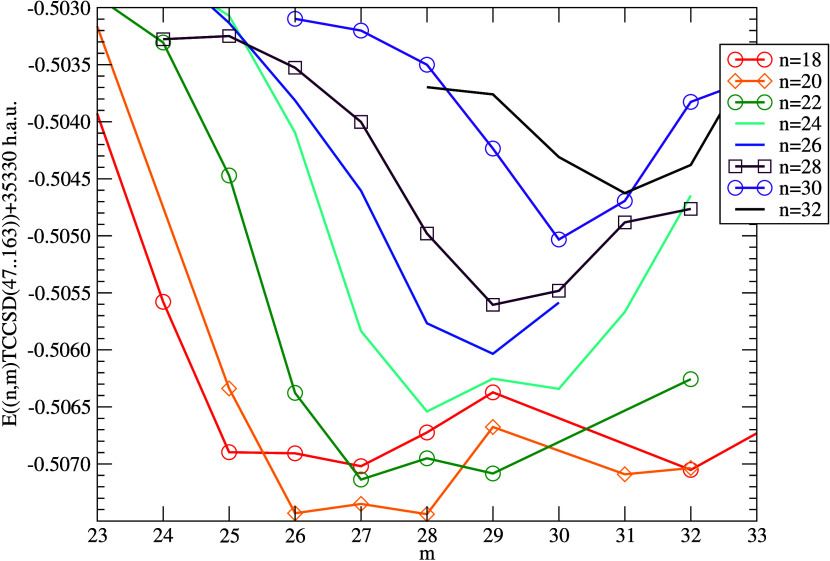
Active space optimization for DMRG(*n*,*m*)-TCCSD single point computation of the NUHFI molecule (*R* = 1.700 Å) in PBE/DZ basis set, bond dimension *M* = 1024, CC orbital space 47–163.

In order to select the DMRG active space, we performed preliminary DMRG calculations with a very large active space (32,98) at lower bond dimensions and for several values of the bond length. Figure 4 shows the results for *r*_N–U_ = 1.5 Å, while the results for other distances can be found in the Supporting Information. The results for A and B spinors form two almost identical blocks, and the bond dimensions 128 and 256 yield very similar results, indicating sufficient accuracy for this purpose. Based on the entropy profiles, we have chosen three active spaces, small (14,15), medium (24,26), and large (32,32). The orbital entropies yield a quantitative indication of how important individual orbitals are, and within the DMRG method, the more one can afford to include in the active space, the better. However, in the TCC method, a too large active space might not be ideal due to the “freezing” of the static correlation in this space, which does not interact with the dynamical correlation effects outside the active space. As shown previously,^20,26^ in the TCC method, a smaller active space may thus yield better results than a bigger one. We have thus also performed an attempt to find the optimum active space for TCCSD, cf. Figure 5, using the DMRG bond dimension *M* = 1024 and CC orbital space restricted to 47–163. As can be seen, the space (20,28) seems to perform best in terms of yielding the lowest TCC energy at the bond distance 1.7 Å. Of course, at a different bond distance, the outcome might be different, and it is clearly not practical to perform such optimization globally. We have thus confined the detailed calculation of the potential energy curves to the three entropy-selected active spaces and this “TCC-optimal” active space.

**Figure 6:**
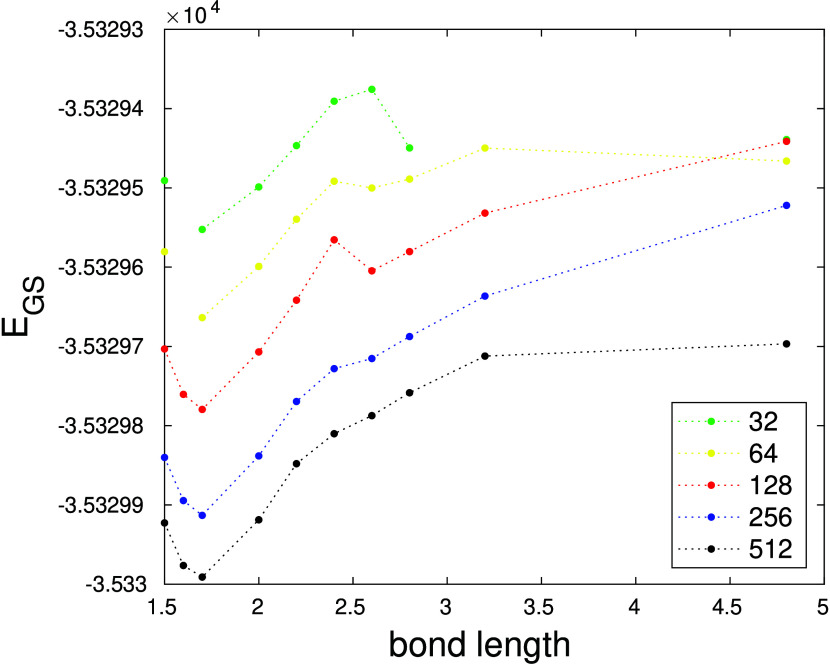
DMRG(32,98) potential energy curves for various bond dimensions.

Besides the entropies, the DMRG(32,98) calculations yielded potential energy curves (at a very coarse bond length grid), which are given in Figure 6. It can be seen that, at the bond dimensions affordable for this very large space, the energies are far from saturation. Since the computational cost of these calculations with a large enough bond length would be intractable, we did not attempt to refine the bond length grid here but performed further DMRG and TCC calculations employing other smaller active spaces selected using either of the orbital entropy criteria (Figure 4).

**Figure 7:**
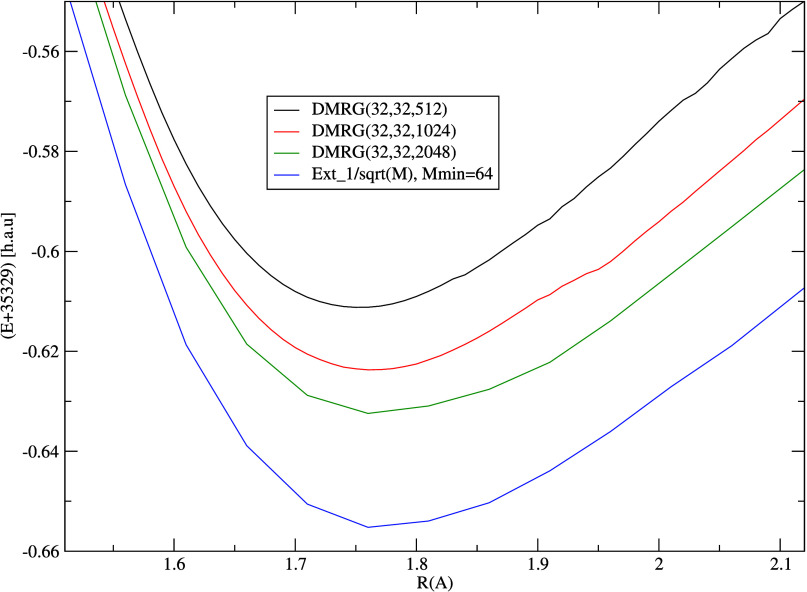
N≡U potential energy curves for DMRG(32,32,M).

We have also investigated the effect of the bond dimension on the DMRG potential energy curves in the largest selected active space (32,32), which are shown in Figure 7. Here, the computational limitation was *M* = 2048, and in spite of this relatively large bond dimension, the DMRG does not seem close to saturation either. We extrapolated DMRG(32,32,*M*) energies to infinite bond dimension *M* using a simple second order polynomial and a three-parameter model:
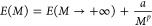
12where *E*(*M*) is the DMRG(32,32,*M*) energy, *E*(*M* → +∞) its extrapolated value, and *a* and *p* are positive fit parameters. *E*(*M*) data have been collected from *M* = 64, 128, 256, 350, 512, 750, 1024, and 2048. (Of course, the bond dimension has a finite maximum value in any finite AO basis, but a large enough to make such an extrapolation sensible.) We have found that the best fit with lowest residual error has been obtained by fixing *p* = −1/2, which is usually applied to fit energy gaps in gapped systems (to which molecules belong), where the correlations decay exponentially. We note that the extrapolation using a general power *p* of eq 12 did not lead to significantly different values and was numerically unstable for *R* > 1.86 Å. The fit via the second order polynomial was very unreliable. A more rigorous fit based on the DMRG truncation error could not be applied here due to the very limited range of the available truncation error data points.

#### IV.B.1 Assessment of TCC in a Small Orbital Space with Respect to Extrapolated DMRG As a Benchmark

As a first step, we decided to compare the performance of TCC with respect to DMRG extrapolated to infinite bond dimension, within a small virtual orbital subspace. We have chosen the DMRG(32,32) space, which corresponds to the range of 66th to 97th Kramers pairs in the CC orbital space. We performed 4c-TCC in several subspaces, namely, (14,15)TCCSD, (20,26)TCCSD, and (24,26)TCCSD, as well as CCSD and CCSD(T) for comparison. The DMRG(32,32) has been extrapolated to the *M* → ∞ limit according to eq 12, which should be equivalent to CASCI(32,32).

**Figure 8:**
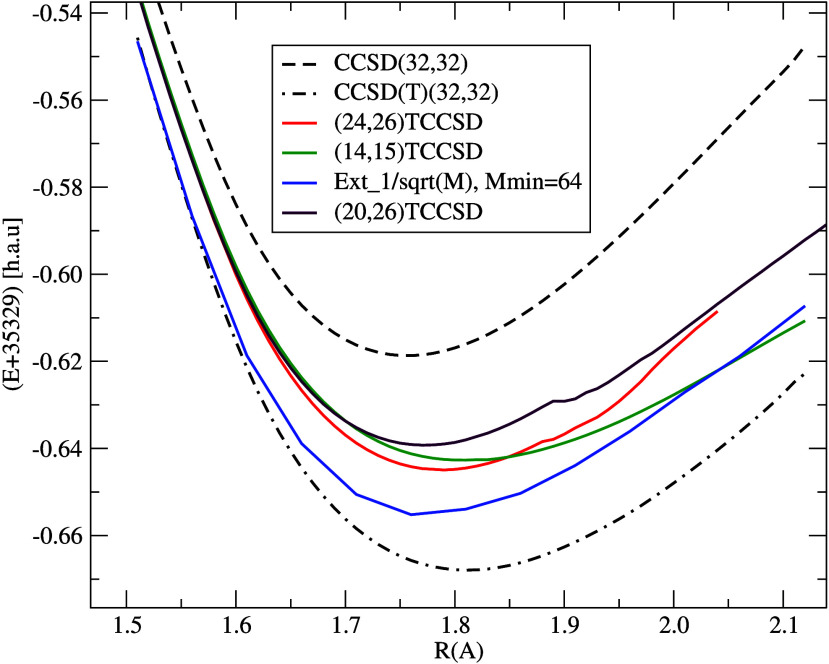
N≡U potential energy curves computed with methods restricted to the (32,32) (active+inactive) orbital space.

As can be seen from Figure 8, the order of energies from different methods is
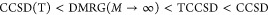
13where all CC methods have amplitude range confined to the DMRG(32,32) subspace. Interestingly, the CCSD(T) curve lies below the extrapolated DMRG benchmark, indicating that the perturbative triples correction overshoots the exact result or the DMRG extrapolation is not able to reproduce the exact (FCI) energy accurately enough. From the potential energy curves in Figure 8, spectroscopic constants have been computed and are listed in Table 2. The results indicate that (24,26)TCCSD provides the closest minimum energy to the extrapolated DMRG(*M* = +∞). The vibrational frequency for (24,26)TCCSD agrees well with CCSD(T) while being about 70 cm^–1^ below the extrapolated DMRG. The (24,26)TCCSD anharmonicity is close to the exptrapolated DMRG, while standard CCSD and CCSD(T) yield lower anharmonicity values. Tailored CC spectroscopic constants are rather sensitive to the active space chosen, which stresses the necessity of appropriate active-space choice for TCCSD. Inside the orbital space (32,32) used as a restriction for both amplitudes and active orbitals, it is not possible to go reasonably much above (24,26), as there will hardly be any amplitudes left. Performing TCC in such a small virtual orbital space, although useful as a sanity check, clearly is not in the spirit of using TCC to capture the dynamic correlation. We attempted a similar test in a larger space corresponding to DMRG(32,98), which was the space used for orbital entropy calculations at low *M*, but it turned out to be computationally too costly.

**Table 2:** Spectroscopic Parameters for the N–U Bond of the NUHFI Molecule in the Dyall-v2z(U,I) and cc-pVDZ (N,H,F) Basis, Restricted to the (32,32) Orbital Space

method	*r*_e_, Å	*E*_min_, a.u.	ω_e_, cm^–1^	ω_e_x_e_, cm^–1^	*Δω*_e_, cm^–1^	*Δω*_e_x_e_, cm^–1^
DMRG(*M* = 1024, d)^c^	1.7591	–35329.62361	1062.42	17.19	–9.35	9.95
DMRG(*M* = 1024, s)^d^	1.7567	–35329.62348	1053.08	19.50	–18.69	12.26
DMRG(*M* = 2048, s)^d^	1.7727	–35329.63243	946.67	8.92	–125.10	1.68
DMRG(*M* = +∞, s)^d^^,^^e^	1.7766	–35329.65525	1006.63	10.04	–65.14	2.80
CCSD	1.7561	–35329.61877	1084.92	5.44	13.15	–1.80
CCSD(T)	1.8110	–35329.66792	929.38	3.17	–142.39	–4.07
(14,15)TCCSD	1.8088	–35329.64278	812.16	2.80	–259.61	–4.44
(20,26)TCCSD	1.7680	–35329.63902	998.56	22.80	–73.21	15.56
(24,26)TCCSD	1.7852	–35329.64465	935.77	10.91	–136.00	3.67

c Dense sampling of distances, with dR = 0.01 Å step. That have been used for all CC methods as well.

d Spare sampling of distances, with dR = 0.05 Å step.

e Extrapolation to infinity M done with respect to *E* = *E*(*M* → + ∞) + *a*/√*M* formula and with set starting with *M*_min_ = 64 and all powers of 2 to *M*_max_ = 2048 together with *M* = 350 and *M* = 750

#### IV.B.2 Potential Energy Curves and Spectroscopic Constants

For the CC and TCC calculations, we have chosen the orbital space comprised by spinors 47–252, as a compromise between accuracy and computational demands. Although it is a severe truncation of the total orbital space in the employed basis set, it already provides a substantial number of external orbitals not in the DMRG active space to allow TCC to capture a significant portion of the dynamic correlation.

**Figure 9:**
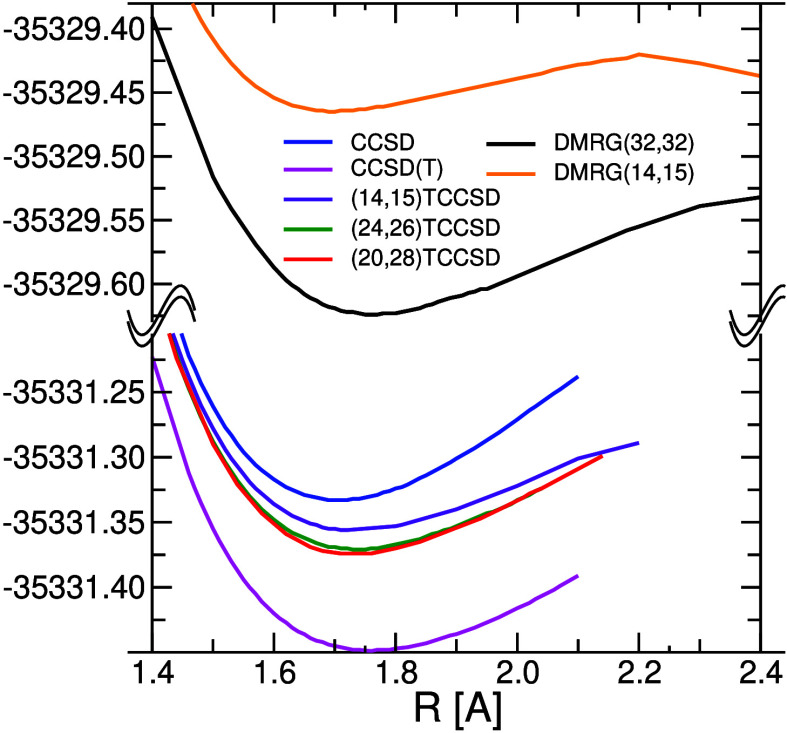
DMRG and CC N≡U potential energy curves. The range of amplitudes has been 47..252 for all CC computations.

**Figure 10:**
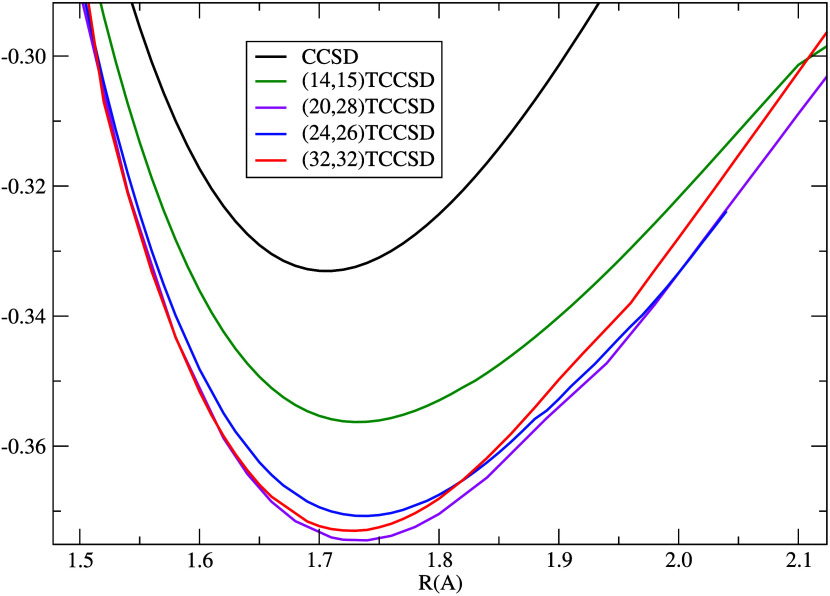
CC and TCC potential energy curves for various active space sizes, amplitude range from the 47th to 252th PBE Kramers pair

The potential energy curves of NUHFI computed by DMRG, CC, and TCC methods are plotted in Figure 9, while a detailed comparison of TCC results is presented in Figure 10 at a finer energy scale. Notice that the TCCSD curves always lie between standard single-reference CCSD and CCSD(T) ones. The CC amplitude equations in TCCSD also converge faster and for larger N–U distances, where ordinary CCSD/CCSD(T) already fails. The better convergence of the tailored CCSD can be explained by two effects: (i) the most problematic amplitudes involving close-lying orbitals around HOMO and LUMO (responsible for the static correlation) are determined by DMRG and fixed, and (ii) there are less amplitudes remaining to be optimized.

Table 3 summarizes our results computed by various methods, as well as data from the available literature. The DMRG and 4c-TCCSD results are presented for several DMRG active spaces, which have been chosen based on orbital entropies and also optimized as described in the previous section. The results for the optimal active space (20,28) have been indicated by boldface. As there are no experimental data for NUHFI molecule so far, only comparison with another computational study^27^ has been possible.

**Table 3:** Spectroscopic Parameters for the N–U Bond of the NUHFI Molecule in the Dyall-v2z(U,I) and cc-pVDZ (N,H,F) Basis^a^

method	*r*_e_, Å	*E*_min_, a.u.	ω_e_, cm^–1^	ω_e_x_e_, cm^–1^	Δω_e_, cm^–1^	Δω_e_x_e_, cm^–1^
4c-DHF-SCF	1.6614	–35329.58205	1280.23	3.59	208.46	–3.65
4c-PBE	1.7408	–35348.15605	1018.67	4.43	–53.10	–2.81
4c-DHF(PBE)	1.6437	–35329.36686	1338.49	3.08	266.72	–4.16
4c-B3LYP	1.7224	–35346.76292	1075.25	3.47	3.48	–3.77
ECP/B3LYP,TZ^b^			1035.0		24.00	
4c-B3LYP,TZ^c^			1065.4		54.40	
4c-DMRG(14,15)^I^	1.7016	–35329.46503	904.28	17.65	–167.49	10.41
4c-DMRG(20,28)^j^	1.7674	–35329.58429	1001.29	4.81	–70.48	–2.43
4c-DMRG(24,26)^k^	1.7547	–35329.54655	847.93	3.78	–223.84	–3.46
4c-DMRG(32,32)	1.7634	–35329.62372	1060.59	7.14	–11.18	–0.10
4c-CCSD^g^	1.7059	–35331.33308	1139.34	3.29	67.57	–3.95
4c-CCSD(T)^g^	1.7604	–35331.44856	973.93	2.99	–97.84	–4.25
4c-(14,15)TCCSD^g^	1.7324	–35331.35627	969.2	7.63	–102.57	0.39
**4c-(20,28)TCCSD**^g^^,^^h^	**1.7314**	**–35331.37442**	**1071.77**	**7.24**	**0.00**	**0.00**
4c-(24,26)TCCSD^d^^,^^g^	1.7353	–35331.06927	1036.53	6.62	–35.24	–0.62
4c-(32,32)TCCSD^g^	1.7250	–35331.37310	1104.49	8.17	32.72	0.93
ECP^f^/CCSD(F12)	1.6819	–642.23964	1149.47	3.58	77.70	–3.66
ECP^f^/CCSD(T)(F12)^d^	1.7061	–642.30227	1095.43	2.69	23.66	–4.55
ECP^f^/CCSD(T*)(F12)^d^	1.7079	–642.31204	1092.03	2.45	20.26	–4.79
ECP^f^/DFT:r2SCAN	1.7114	–643.73867	1061.35	3.38	–10.42	–3.86
ECP^f^/DFT:mpsts-noa2	1.7056	–643.51394	1077.54	3.34	5.77	–3.90

a The parts of potential energy curves delimited by a maximal N–U distance *R*_max_ = 2.100 Å and a minimal *R*_min_ corresponding to the same energy as at *R*_max_ have been used for the fit and calculation of spectroscopic constants, unless indicated otherwise below.

b Scalar quasi-relativistic computation. From ref (27).

c Computation From ref (27), harmonic value, four-component Hamiltonian.

d *R*_max_ = 2.040 Å.

f Scalar quasi-relativistic, with def2-TZVPP AO basis, computed using Turbomole V7.6.^62,70^

g 4c-CC and almost all 4c-TCC computations have been done with amplitude range 47–252.

h The bold text corresponds to the optimal active space for the 4c-TCCSD method selected by minimization of 4c-TCCSD energy at *R* = 1.700 Å (see text for more details).

I Bond dimension *M* = 2048, number of sweeps = 41.

j *R*_max_ = 1.850 Å.

k *R*_max_ = 1.880 Å.

Compared to all correlated methods, 4c-DHF-SCF underestimates the bond length and anharmonicity, while it overestimates the vibrational frequency. DHF computed from the PBE orbitals (employed as a reference for post-HF methods in this work) shows an even stronger tendency in this direction, unsurprisingly demonstrating the importance of the correlation effects.

We performed also a 4c-B3LYP calculation in the basis set employed in this work and compared it to the B3LYP vibrational frequencies in a larger basis set from ref (27). There is no dramatic effect of the basis set size on the vibrational frequency, which supports the use of our basis set of choice. (As we had to truncate the virtual orbital space for CC calculations, the use of a larger AO basis for our calculations would not be sensible anyway.)

Next part of Table 3 shows DMRG results in several active spaces. In the smallest one (14,15), the bond length is rather close to the DHF value, while it becomes larger for the larger spaces. The DMRG vibrational frequencies lie below 1000 cm^–1^ except for the (20,28) and (32,32) spaces, while the anharmonicities differ over a wide range. Particularly the anharmonicity from the smallest DMRG space (14,15) is an outlier. The reason might be that these spaces are rather small and addition of a few more orbitals has thus a large effect. However, as can be seen on the next lines of the table, the 4c-TCCSD method, although based on DMRG, yields anharmonicity values in a rather narrow range, even for the small space, where DMRG value was extremely large.

The 4c-TCCSD method yields similar values of the equilibrium distance (around 1.73 Å), regardless of the active space. The vibrational frequency is more sensitive on the active space choice, but except for the smallest space (14,15), it always lies above 1000 cm^–1^. Anharmonicities are again less sensitive to the active space, being all around 7 cm^–1^. Compared to standard single-reference 4c-CCSD and 4c-CCSD(T), the 4c-TCCSD equilibrium distance and vibrational frequency lies between the 4c-CCSD and 4c-CCSD(T) values. This might be explained by the fact that these properties are determined by the PEC around equilibrium, where the multireference character is still weak and 4c-TCCSD can capture more of the dynamic correlation than 4c-CCSD but less than 4c-CCSD(T). On the other hand, the 4c-TCCSD anharmonicity is approximately twice as large than the 4c-CCSD or 4c-CCSD(T) values, which are similar. Since already the DMRG method alone yielded larger anharmonicities, although widely varying in dependence on the active space, one can deduce that the strong correlation is more important for the anharmonicity, in line with the fact that the multireference character of the system grows with the elongation of the N–U bond. Single-reference methods like CCSD or CCSD(T) thus probably underestimate the anharmonicity. It is possible that DMRG or 4c-TCCSD might on the other hand overestimate it, but for the NUHFI molecule we unfortunately do not have an experimental reference.

Finally, we performed also scalar ECP quasirelativistic calculations with CCSD and CCSD(T) in their explicitly correlated F12 versions to better capture the dynamic correlation and advanced DFT methods (r2SCAN is regularized-restored Strongly Constrained and Appropriately Normed meta-GGA functional;^71^ mpsts-noa2 is (modified) functional of Perdew, Staroverov, Tao, and Scuseria (PSTS-LMF)^72,73^) implemented in the Turbomole code,^62,70^ using a larger def2-TZVPP basis set. The bifurcation of the SCF solution in this case occurred at a larger bond length; therefore, we were able to obtain smooth curves for the post-HF methods in a large enough range to compute the spectroscopic constants. All these methods yield slightly shorter bond lengths than the 4c-TCCSD methods, which might be due to the different basis or due to different treatment of the relativity, but the vibrational frequencies at the CCSD(T)-F12 level are close to the 4c-TCCSD results discussed above. The DFT methods yield actually very similar results to those of 4c-CCSD(T). Again, all of these methods yield anharmonicities smaller than 4c-TCCSD, which is probably due to their single-reference character.

## V Conclusions

We have implemented the relativistic tailored coupled cluster method (4c-TCCSD), in combination with the Budapest DMRG and DIRAC codes. The method is capable of treating relativistic, strongly correlated systems and includes a significant portion of the dynamical correlation. The present implementation is fully general, applicable to polyatomic molecules, and is not limited to a particular molecular symmetry double group.

We have applied the method to the study of the stretch of the nitrogen–uranium bond in NUHFI and NUF_3_ molecules. This bond can be viewed as a relativistic analogue of the triple bond in N_2_, which is well-known for its multireference character at stretched bond lengths. The chiral NUHFI molecule is also interesting as a candidate for measurable electroweak parity violating effects, while for NUF_3_, experimental vibrational frequency is available.

It turned out that the choice of the molecular spinors (orbitals) for the post-HF calculations of these molecules is nontrivial, as the DHF-SCF solutions for both molecules exhibit a bifurcation to two branches around 2 Å. Relativistic CASSCF (or DMRG-SCF) calculation was not possible for the low-symmetry NUHFI molecule with presently available codes, so we have employed PBE Kohn–Sham spinors as the molecular spinor basis.

The 4c-TCCSD method with optimal active space yielded for NUF_3_ vibrational frequency about 17 cm^–1^ above the experimental value, while the anharmonicity was in line with an estimate based on an experimental value for similar molecules. This agreement was considerably better than the performance of DMRG or single-reference 4c-CCSD alone. For both molecules, the 4c-TCCSD method yielded bond lenghts and vibrational frequencies between the values from single-reference 4c-CCSD and 4c-CCSD(T) methods, indicating that 4c-TCCSD is able to capture more dynamical correlation than 4c-CCSD but less than 4c-CCSD(T). 4c-TCCSD yielded larger anharmonicity values than the single-reference CC methods, which can be attributed to the better description of the potential energy curves at larger distances, where the strong correlation plays a bigger role. The 4c-TCCSD method thus offers benefits compared to single-reference CC methods; however, for future applications the availability of relativistic 4c-CASSCF and/or DMRG-SCF without symmetry restrictions will be important.

## Appendix. Choice of the DFT Functional Employed to Obtain the Molecular Spinor Basis

Since the DHF-SCF suffers from a bifurcation of the potential energy curve and CASSCF was not technically possible for the nonsymmetric molecule, we decided to employ DFT Kohn–Sham spinors as a MO basis for the post-HF calculations. In order to select a functional, we performed a small scalar quasirelativistic ECP/DFT study of six chosen systems with uranium bonded to terminal nitrogen, where the experimental value for ω_e_ could be found (Table 4). We employed three common XC functionals (PBE, BLYP, and B3LYP) and def-TZVPP basis set as implemented in the Turbomole program.^62,70^

**Table 4:** DFT Functional Selection for Set of Molecules Containing N≡U Bond, N≡U Bond Stretching Frequency ω_e_ in cm^–1^

system	exper.	ref	B3LYP	BLYP	PBE
UN^+^^a^	1083	(68)	1094	1025	1058
UN	1010	(69)	1038	977	1010
NUO^+^	1017	(69)	1182	1097	1031
NUN^b^	1051	(69)	1116	1054	1083
NUF_3_	938	(61), (69)	1042	934	973
NUNH	967	(69)	1038	979	1013
MAE^d^			74	32	26
MQE^e^			90	43	30
NUHFI	n.a.^c^		1073	974	1011

a For this molecular ion, also experimental anharmonicity ω_e_*x*_e_ = 5.3 cm^–1^ has been determined.^68^

b Antisymmetric stretch (σ_u_), visible in IR spectra.

c To our best knowledge, NUHFI molecule has not been synthesized yet; there are thus no experimental data.

d Mean absolute error.

e Square root of mean of squares of errors.

From the comparison of the computed vibrational frequencies with the experimental ones, it can be concluded that the best performing functional is PBE. We have thus selected it as the source of molecular spinors for the post-HF calculations.

## Supporting Information Available

Files for frequency recomputation (ZIP)
Optimized geometries, assessment of XC functionals, ECP computational details, visualization of active molecular orbitals, TCC active space optimization, results in MP2 NO basis, orbital entropy figures (PDF)
