# Supplementary material for: DMRG-Tailored Coupled Cluster Method in the 4c-Relativistic Domain: General Implementation and Application to the NUHFI and NUF3 Molecules
Source: J Chem Theory Comput. 2024 Oct 9;20(20):8862–75. doi: 10.1021/acs.jctc.4c00641 (PMC11500409; doi:10.1021/acs.jctc.4c00641)
Supplement: Supplementary file 2 — ct4c00641_si_002.pdf [file ct4c00641_si_002.pdf]

Supplementary Information

DMRG-tailored coupled cluster method in the 4c-relativistic domain: General implementation and application to the NUHFI and NUF_3 molecules

Jakub Višňák[1,2,3], Jan Brandejs[1,2,4], Mihály Maté[5,6], Lucas Visscher[7], Örs Legeza[5,a)] and Jiří Pittner[1,b)]

[1)] J. Heyrovský Institute of Physical Chemistry, Academy of Sciences of the Czech Republic, v.v.i., Dolejškova 3, 18223 Prague 8, Czech Republic

[2)] Faculty of Mathematics and Physics, Charles University, Ke Karlovu 3, 12116 Prague, Czech Republic

[3)] Middle East Technical University, Üniversiteler Mahallesi, Dumlupınar Bulvarı No:1, 06800 Çankaya Ankara, Türkiye

[4)] Faculty of Science, Humanities and Education, Technical University of Liberec, Studentská 1402/2 461 17, Liberec, Czech Republic

[5)] Strongly Correlated Systems „Lendület" Research Group, Institute for Solid State Physics and Optics, MTA Wigner Research Centre for Physics, H-1121 Budapest, Konkoly-Thege Miklós út 29-33, Hungary

[6)] Department of Mathematics, Technical University of Munich, Boltzmannstr. 3, 85748 Garching, Germany

[7)] Amsterdam Center for Multiscale Modeling, VU University Amsterdam, NL-1081 HV Amsterdam, Netherlands

[a)] Electronic mail: legeza.ors@wigner.mta.hu

[b)] Electronic mail: jiri.pittner@jh-inst.cas.cz

Optimized geometries

element	x [A]	y [A]	z [A]
N	0.0000000	0.0000000	1.7097000*
U	0.0000000	0.0000000	0.0000000
H	-1.8902788	0.0000000	-0.5369563
F	0.3904333	0.8214924	-1.7822566
I	0.2824494	-2.6997796	-1.0041165

Tab. S1: Cartesian coordinates: ECP/def-TZVPP/B3LYP (in Turbomole V7.6) optimised NUHFI molecule. The data points have been produced by varying the z-coordinate of nitrogen (N) denoted by *.

element	x [A]	y [A]	z [A]
U	0.000000	0.000000	0.000000
N	0.000000	0.000000	1.753000*
F	1.722815	0.000000	-1.101786
F	-0.861408	-1.492002	-1.101786
F	-0.861408	1.492002	-1.101786

Tab. S2: Cartesian coordinates from - NUF_3 molecule (C_{3v} point group). The data points have been produced by varying the z-coordinate of nitrogen (N) denoted by *. This geometry has been adopted from CASPT2 computation found in the literature [Atkinson2018].

XC functionals used for 4c-DFT (NUHFI)

For the 4c-DFT computation of NUHFI (in DIRAC21 using Dyall.v2z and cc-pDVZ atomic basis sets, as described in detail in article computationals) in its above-mentioned optimised geometry and with N-U bond prolonged multiple XC functionals have been used. Some notable examples are presented in Fig. 1, showing the dissociation curve along the N-U bond (R(A) on the vertical axis corresponds to its length in Angstroems). „HF from [X], DZ“ corresponds to a single Hartree-Fock determinant energy (evaluated by the CCSD module of DIRAC) using Kohn-Sham bispinors („KS orbitals“) as orbitals. Since KS orbitals have been later on used as MO basis set for following DMRG, CCSD, TCCSD and CCSD(T) computations, only those having a reasonably physical behaviour of single determinant (or Hartree-Fock, HF) energy with increased N-U distance (R(A)) are to be used.

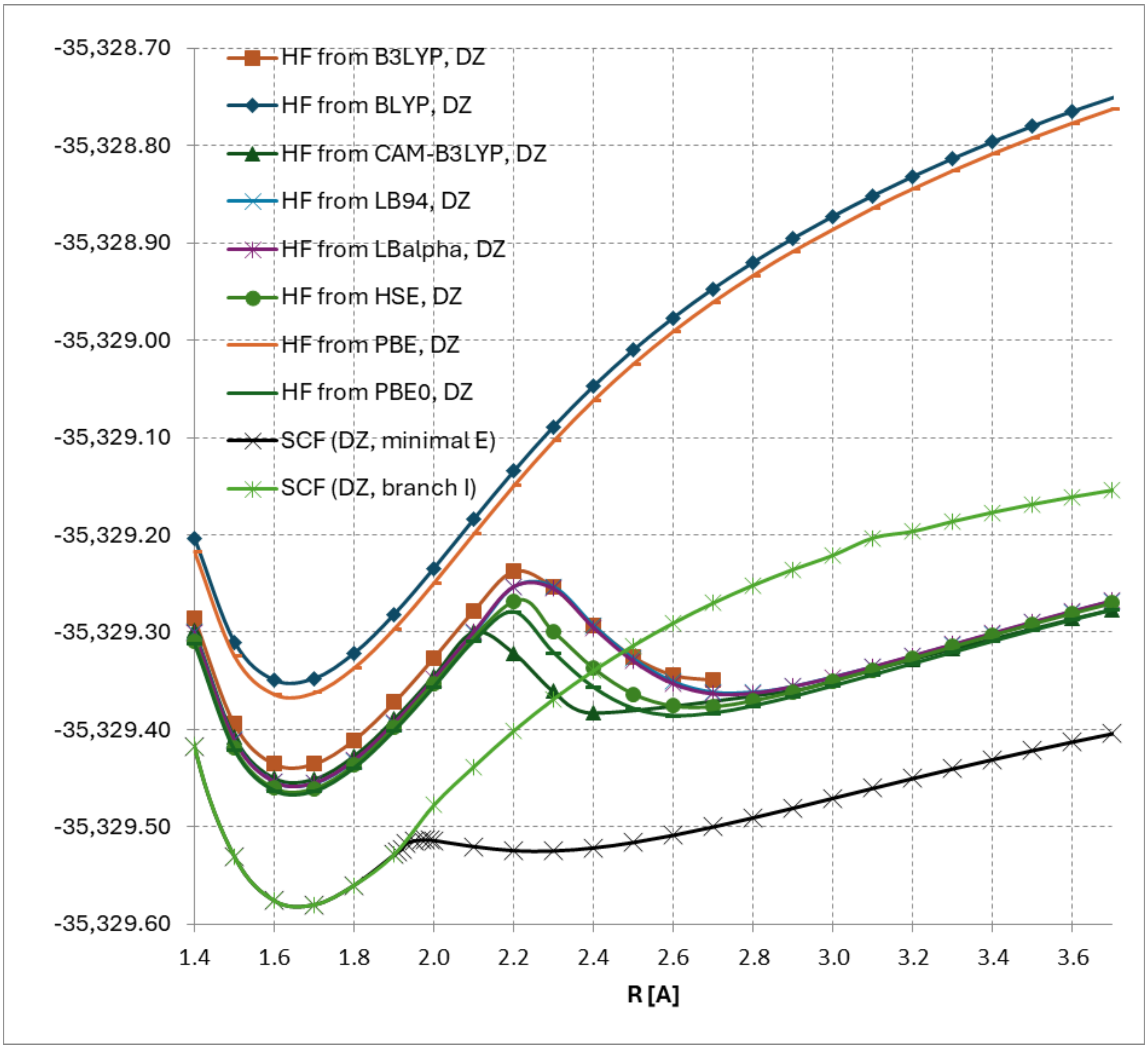

Fig. S1: SCF (both branches) and HF from Kohn-Sham orbitals corresponding to different DFT functionals

The dissociation Hartree-Fock curves corresponding to screened-hybrid, hybrid or meta-GGA functionals’ Kohn-Sham orbitals show an unphysical local maximum between R = 2.10 A and R = 2.25

A (position depending on the functional type). Behaviour resembling the „minE“ (or „minimal E“) branch of the SCF solution curve.

However, HF curves corresponding to lower rung functionals (GGA) show physical dissociation curves without local maxima; among them, respecting the variational principle, we have chosen PBE over BLYP.

ECP computation details

For comparison to 4c-HF, 4c-DFT, 4c-DMRG, 4c-CCSD, 4c-CCSD(T) 4c-TCCSD several scalar quasirelativistic computations have been done in Turbomole V7.4 (TM) using effective-core pseudopotentials. Explicitly correlated (F12) computations, which are available in the program under the RIJ-approximation, have been performed as well. in orbital basis sets (def-TZVPP, H,N,F: [Weigend2005a], I: [Weigend1998], U:([CaoDolg2004] where three g functions were added with exponents 0.37, 1.4, 4.5)). Explicitly correlated computations in TM require several auxiliary basis sets (of def-TZVPP quality) denoted cbas (Auxiliary basis sets for RI-MP2 and RI-CC, elements H–Ar), jbas (Auxiliary basis sets for RI-J in HF/DFT(Coulomb fitting)), jkbas (Auxiliary basis sets for RI-K in HF/DFT(Exchange fitting)) and cabs (Complementary auxiliary basis sets, for the F12 methods), which are listed for the respective elements in the table below.

Element	cbas	jbas	jkbas	Cabs
N	[Weigend1998]	[Eichkorn1997]	[Weigend2002]	[Weigend1998]
U	[Weigend1998]*	[Weigend2006]*	[Weigend2008]*	[Hellweg2007]*,**
H	[Weigend1998]	[Eichkorn1997]	[Weigend2002]	[Weigend1998]
F	[Hellweg2007]	[Eichkorn1997]	[Weigend2002]	[Weigend1998]
I	[Weigend1998]	[Eichkorn1997]	[Weigend2008]	[Hellweg2007]

Tab. S3: Auxiliary basis sets.

Effective core potentials for uranium and iodine have been used as standard built-in Turbomole („def-ecp“, I:[Schwerdtfeger1989], U:[CaoDolg2004]). The iodine jbas „universal-ecp-28“.

* Due to lack of data, basis set for the heaviest atom available (Rn) has been used instead. Four-fold increase in exponents (added by linear interpolation of their logarithms) did not led to change of vibrational frequency highr than 4 cm^{-1}.

** Due to lack of data, cbas have been used instead of cabs here.

ECP/SCF computations for NUF_3

Both NUHFI and NUF_3 potential energy surface cuts along the N-U coordinate did show branching of SCF solution around R = 2 A in both 4c- and ECP computations, but in the case of NUHFI, the ECP/SCF branching point have been well above R > 2.1 A and thus not important for spectroscopic constants evaluation (where we limited ourselves to part of curve below R = 2.1 A).

The case of ECP/SCF energies along the N-U bond stretch in NUF3 (R is the N-U bond length in Angstroems) is presented below. The NUF_3 has been considered as C_{3v} symmetric with reference geometry from [Atkinson2018].

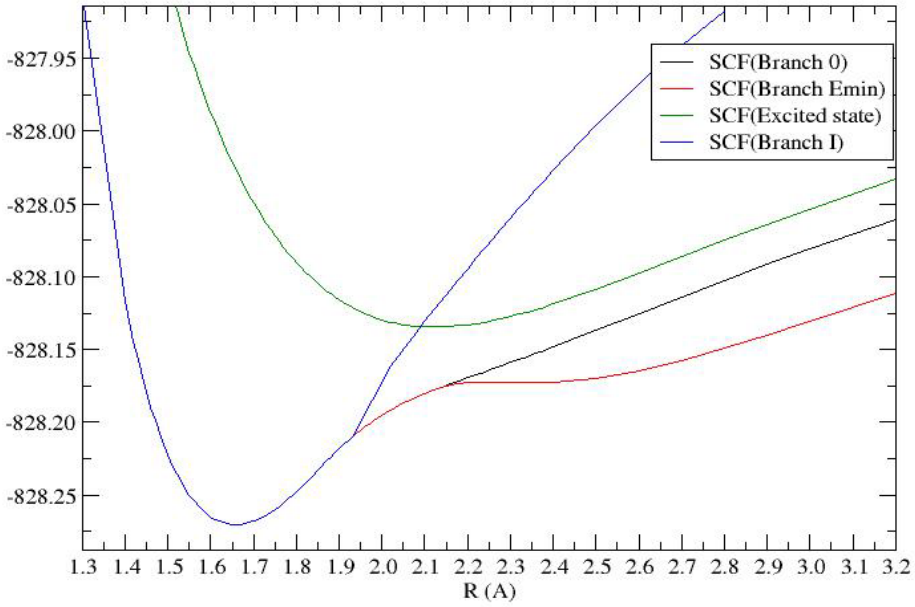

Fig. S2: ECP/SCF potential energy surface cut (along N-U stretching coordinate) curves for NUF_3. For R ≤ 2.1 A, there are two branches for the ground state.

Each branch's molecular orbitals have been used to compute corresponding ECP/CCSD(F12), ECP/CCSD(T)(F12) and ECP/CCSD(T*)(F12) curves (branching effect for the latter is depicted in Fig. S3) and evaluate spectroscopic constants (see Table III in the main article).

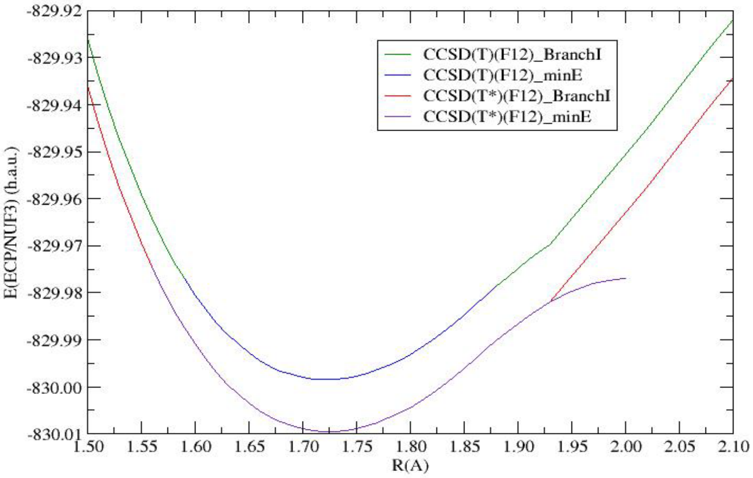

Fig. S3: Parts of ECP-based potential energy cuts along N-U bond stretch used for evaluation of spectroscopic constants in Tab. III of the main article

PBE molecular bispinors

NUF_3 molecule

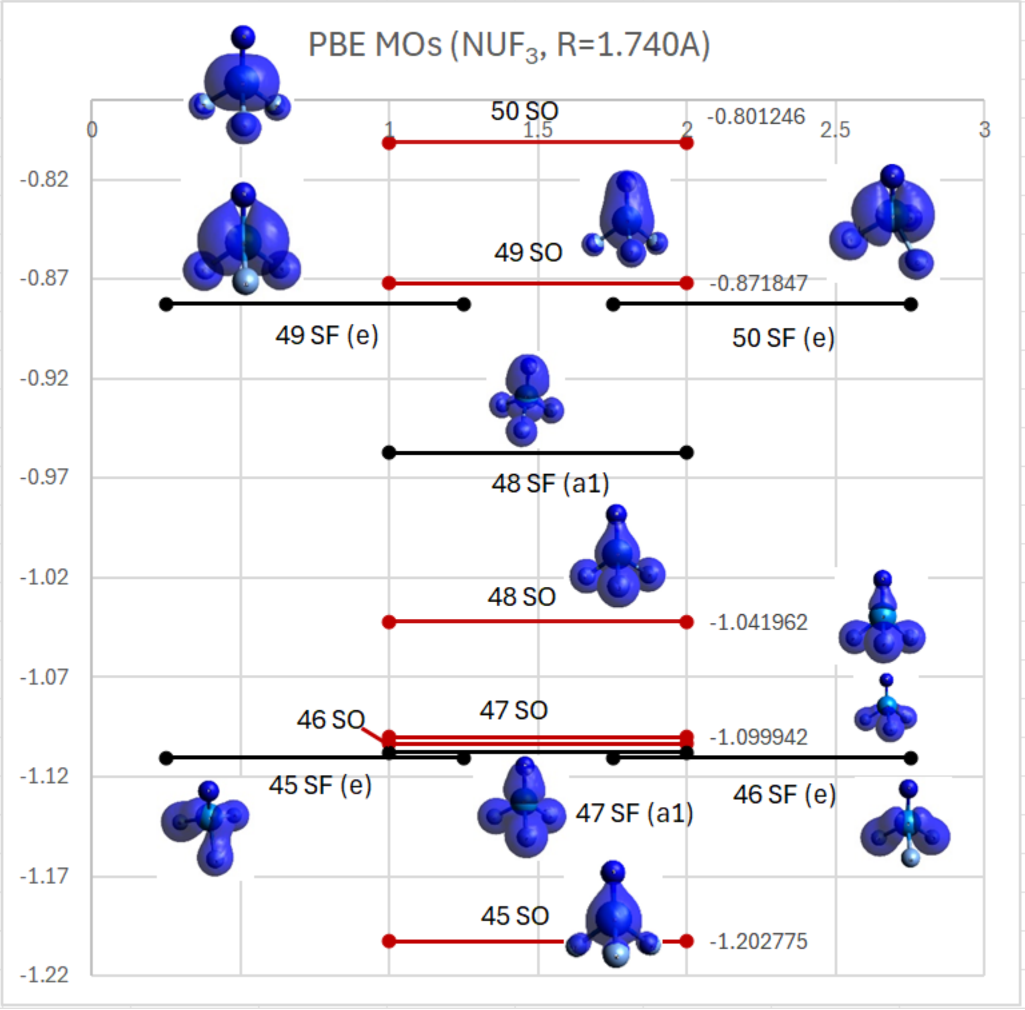

Fig. T1: NUF_3 orbital isodensities for Kramer pairs 45 to 50.

Densities of any of the two 4c-wave functions constituting the Kramer pair are plotted, isodensity = 0.002 if not specified otherwise. SF = spin-free computation (4c- Dirac-Coulomb Hamiltonian, but all spin-dependent terms projected out, energy levels plotted in black), SO = full Coulomb-Dirac 4c-treatment (energy levels plotted in red and energies written aside to them). C_{3v} point group has irreducible representations a1, a2 and two-dimensional e; they can be used as labels in the spin-free case and are indicated in parentheses.

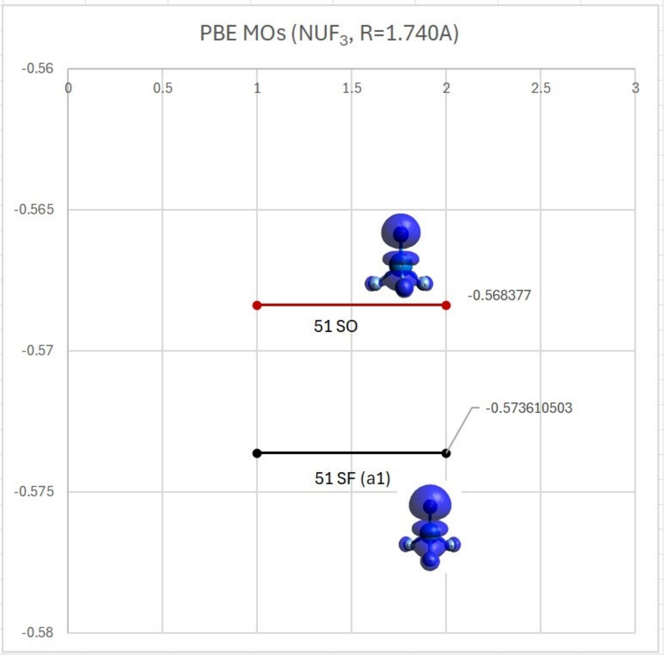

Fig. T2: NUF_3 orbital isodensities for Kramer pair 51.

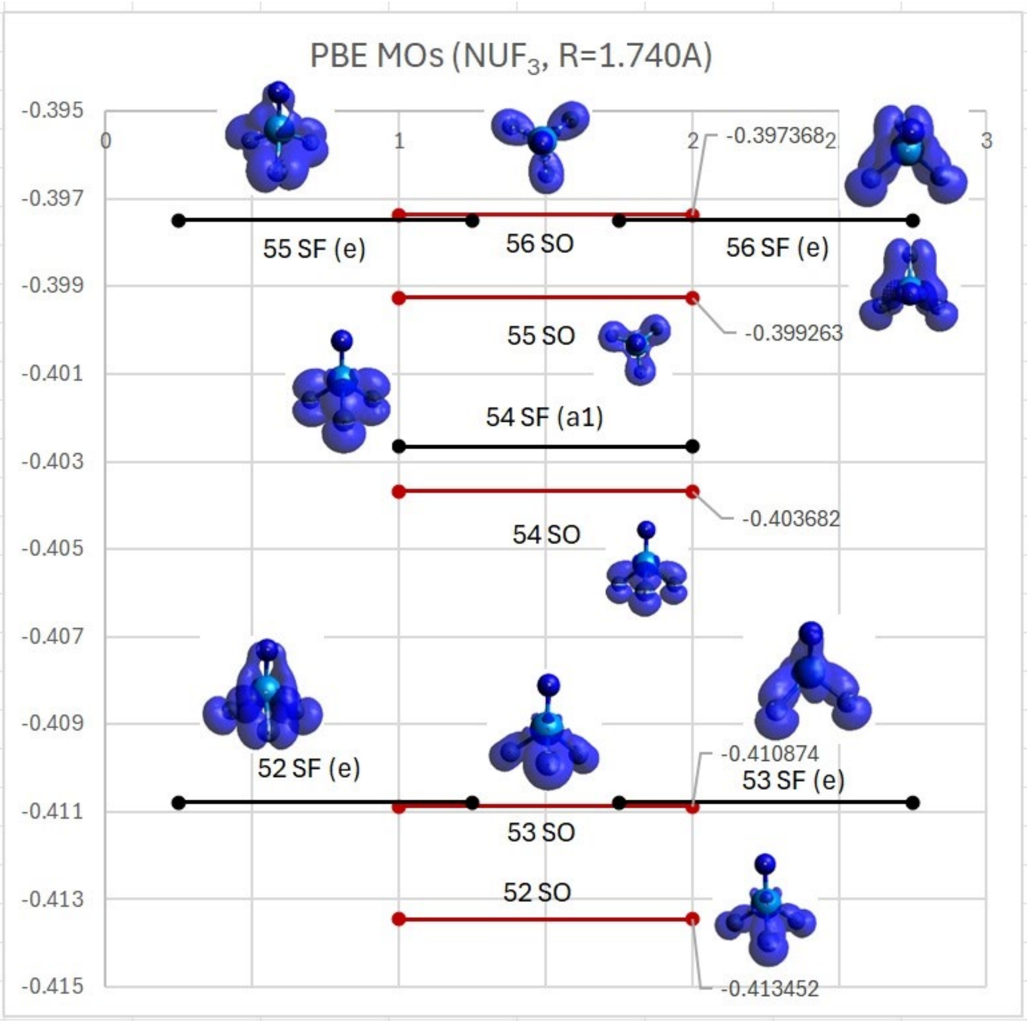

Fig. T3: NUF_3 orbital isodensities for Kramer pairs 52 to 56.

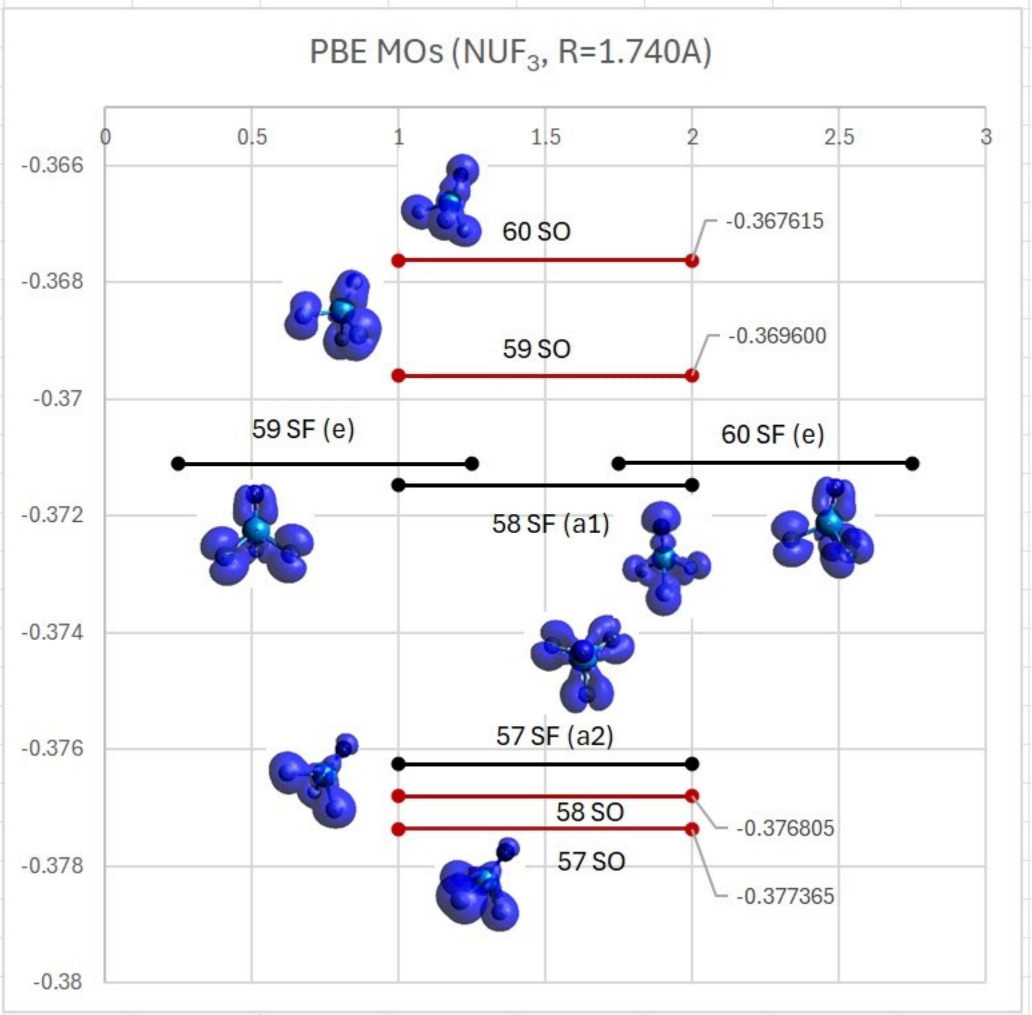

Fig. T4: NUF_3 orbital isodensities for Kramer pairs 57 to 60.

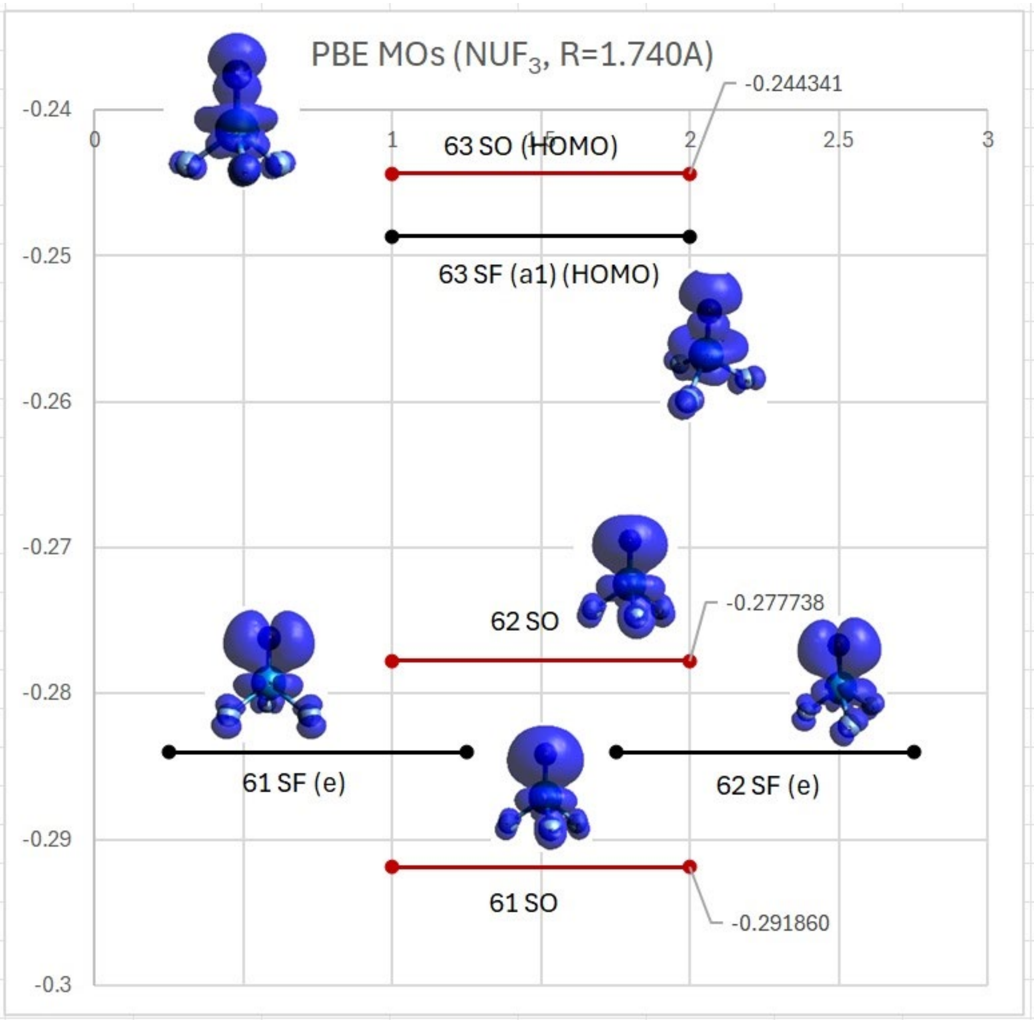

Fig. T5: NUF_3 orbital isodensities for Kramer pairs 61 to 63.

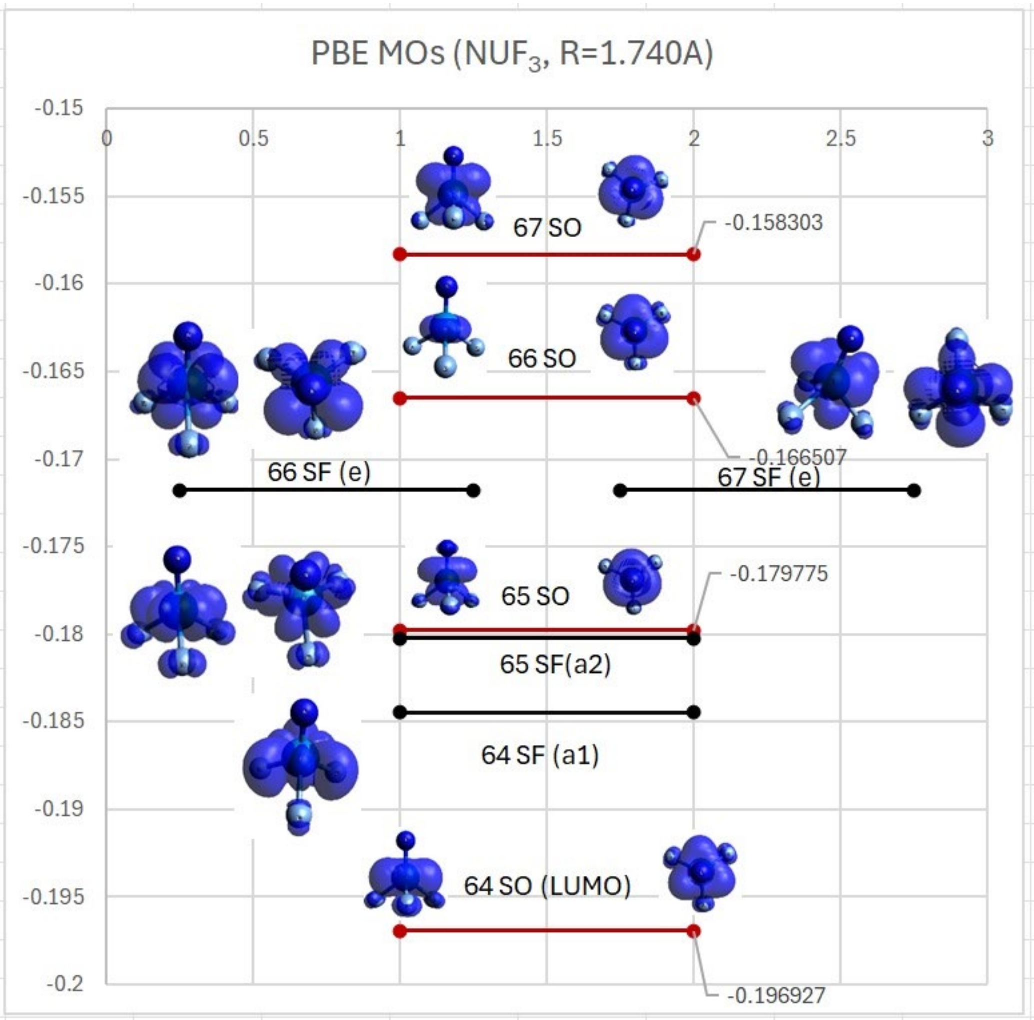

Fig. T6: NUF_3 orbital isodensities for Kramer pairs 64 to 67.

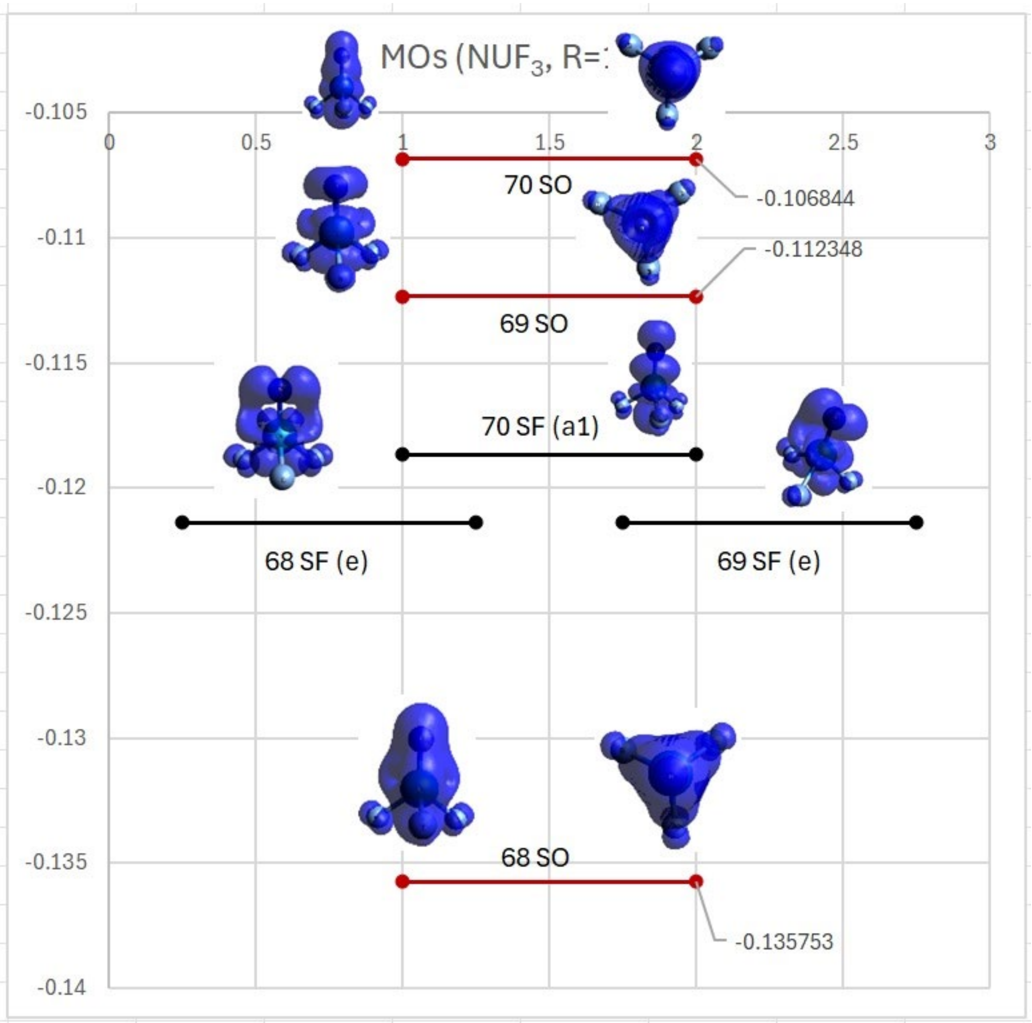

Fig. T7: NUF_3 orbital isodensities for Kramer pairs 68 to 70.

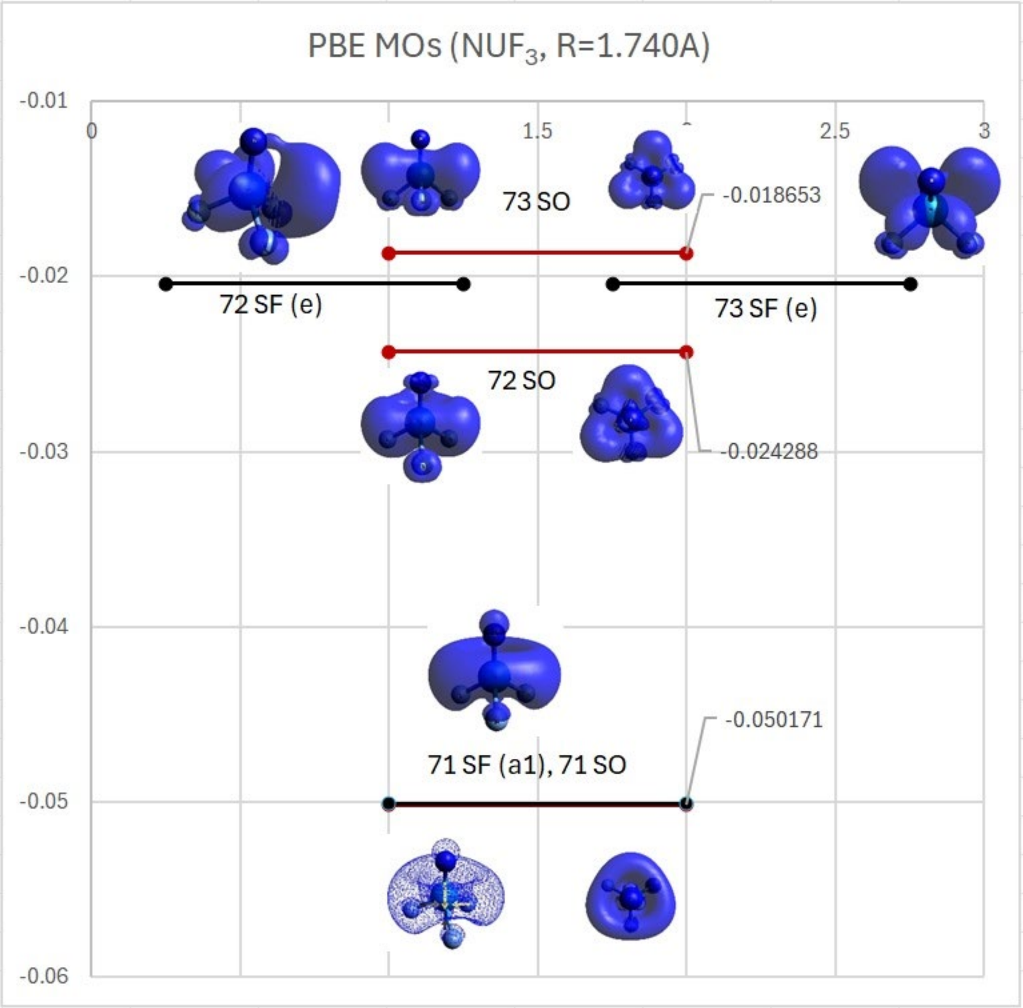

Fig. T8: NUF$_3$ orbital isodensities for Kramer pairs 71 to 73.

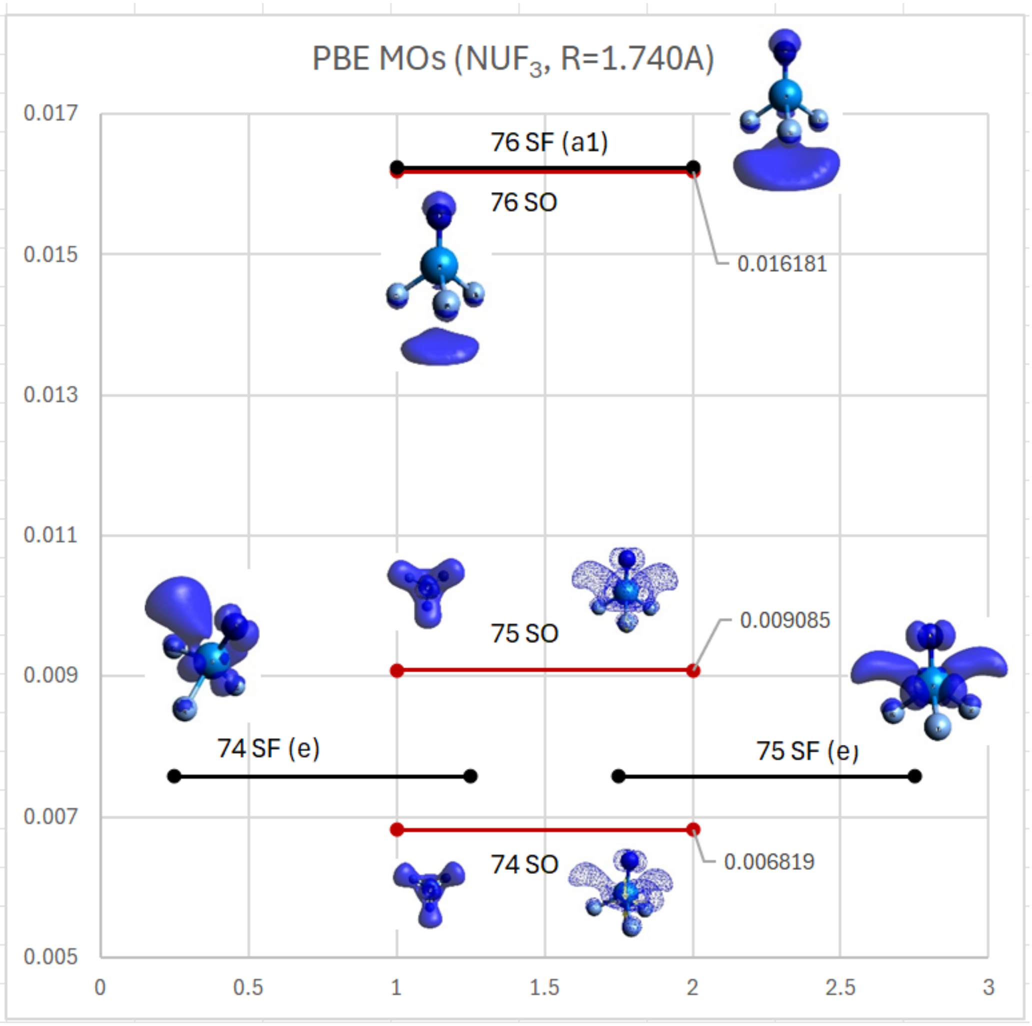

Fig. T9: NUF_3 orbital isodensities for Kramer pairs 74 to 76.

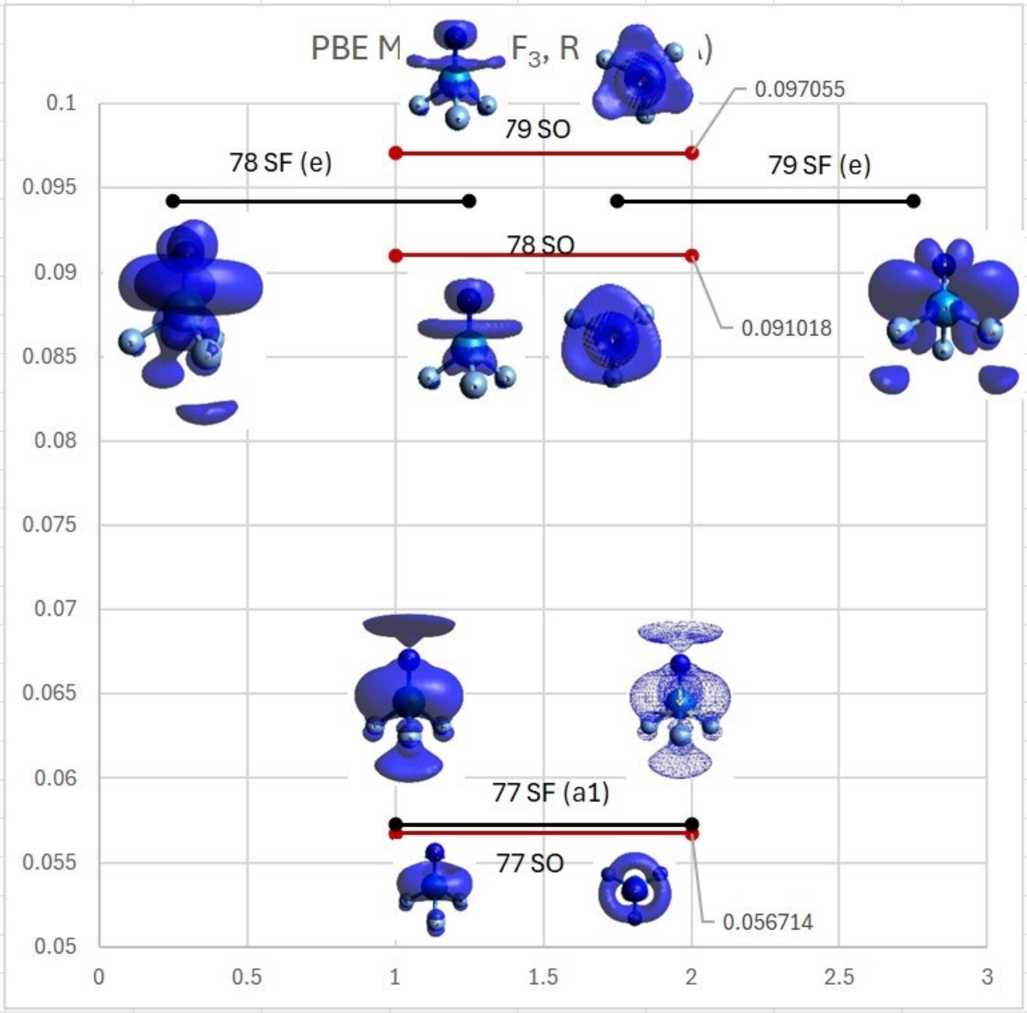

Fig. T10: NUF_3 orbital isodensities for Kramer pairs 77 to 79.

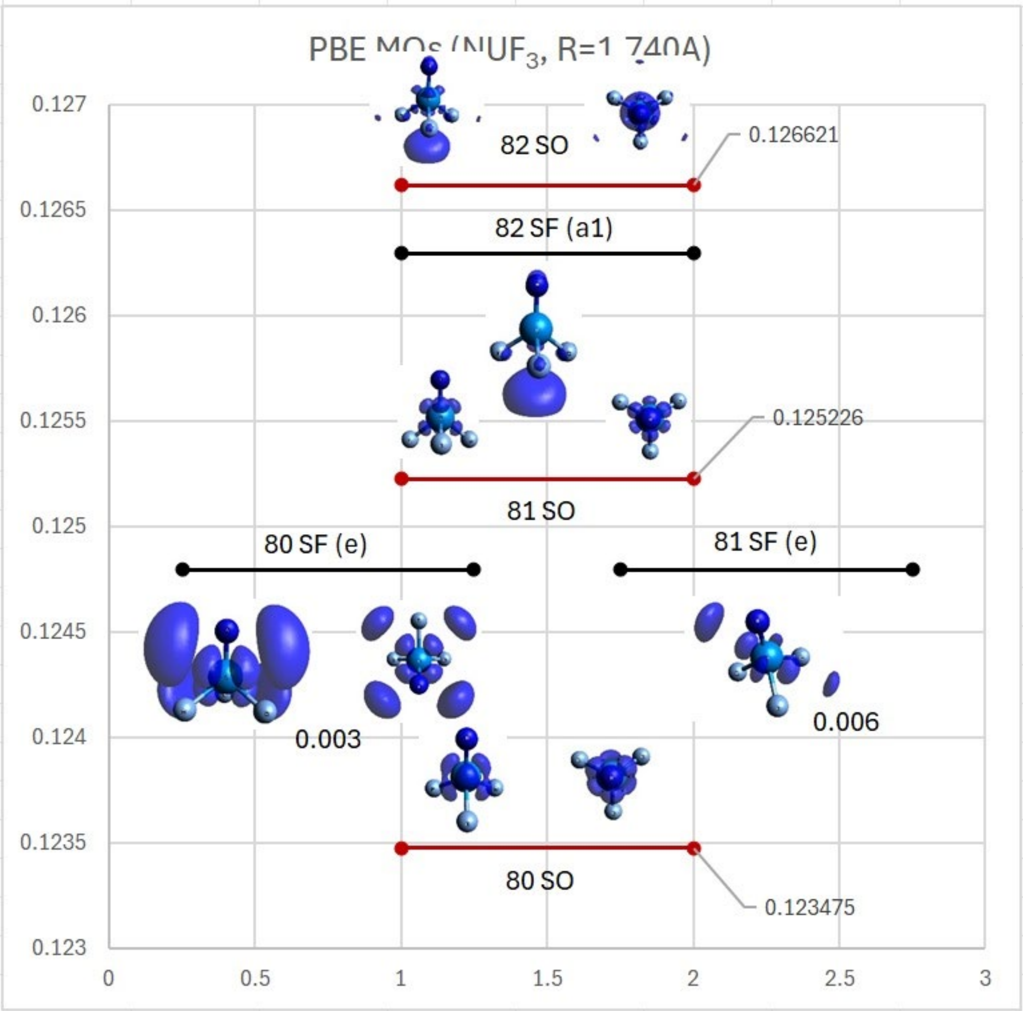

Fig. T11: NUF_3 orbital isodensities for Kramer pairs 80 to 82.

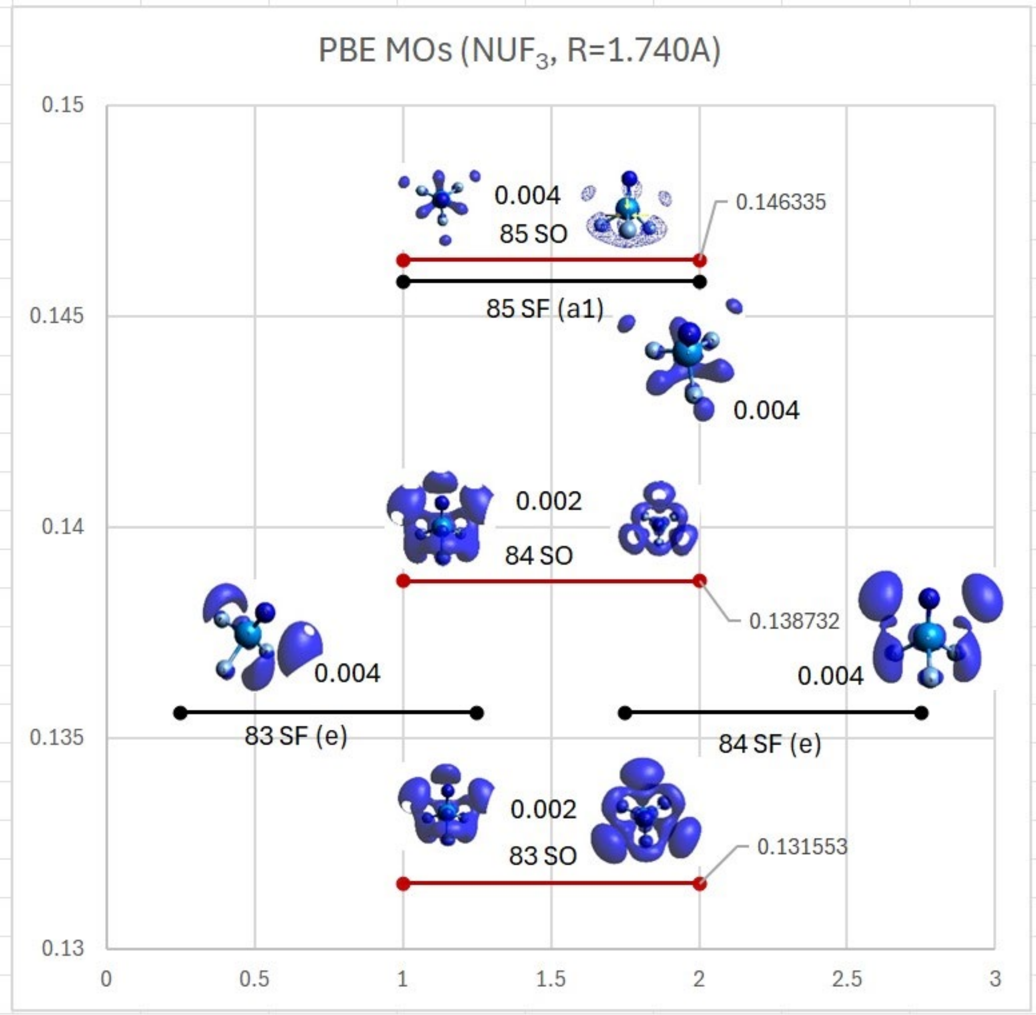

Fig. T12: NUF_3 orbital isodensities for Kramer pairs 83 to 85.

The following figures show only the „SO“ case (corresponding to the article), the value after the Kramer pair index is the isodensity of the plot.

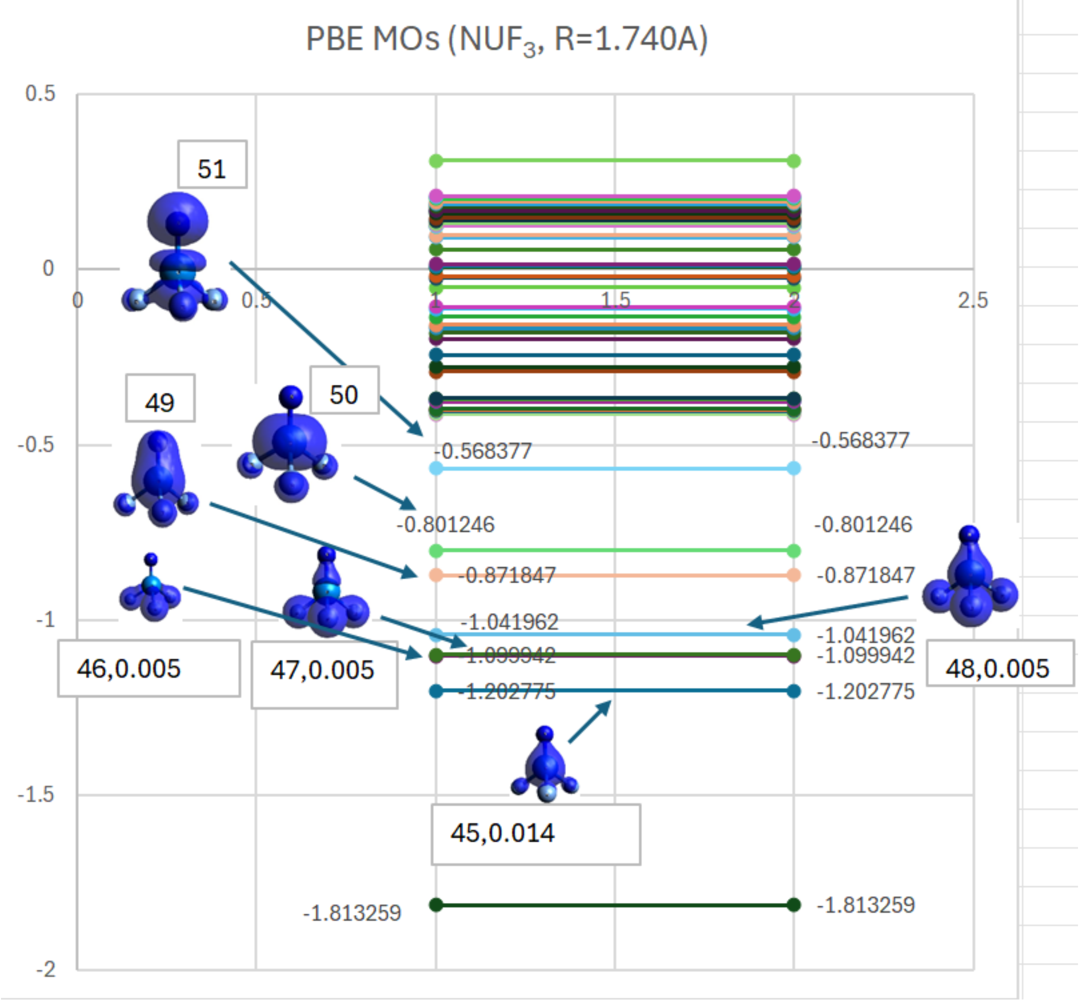

Fig. T13: NUF_3 orbital isodensities for Kramer pairs 45 to 51.

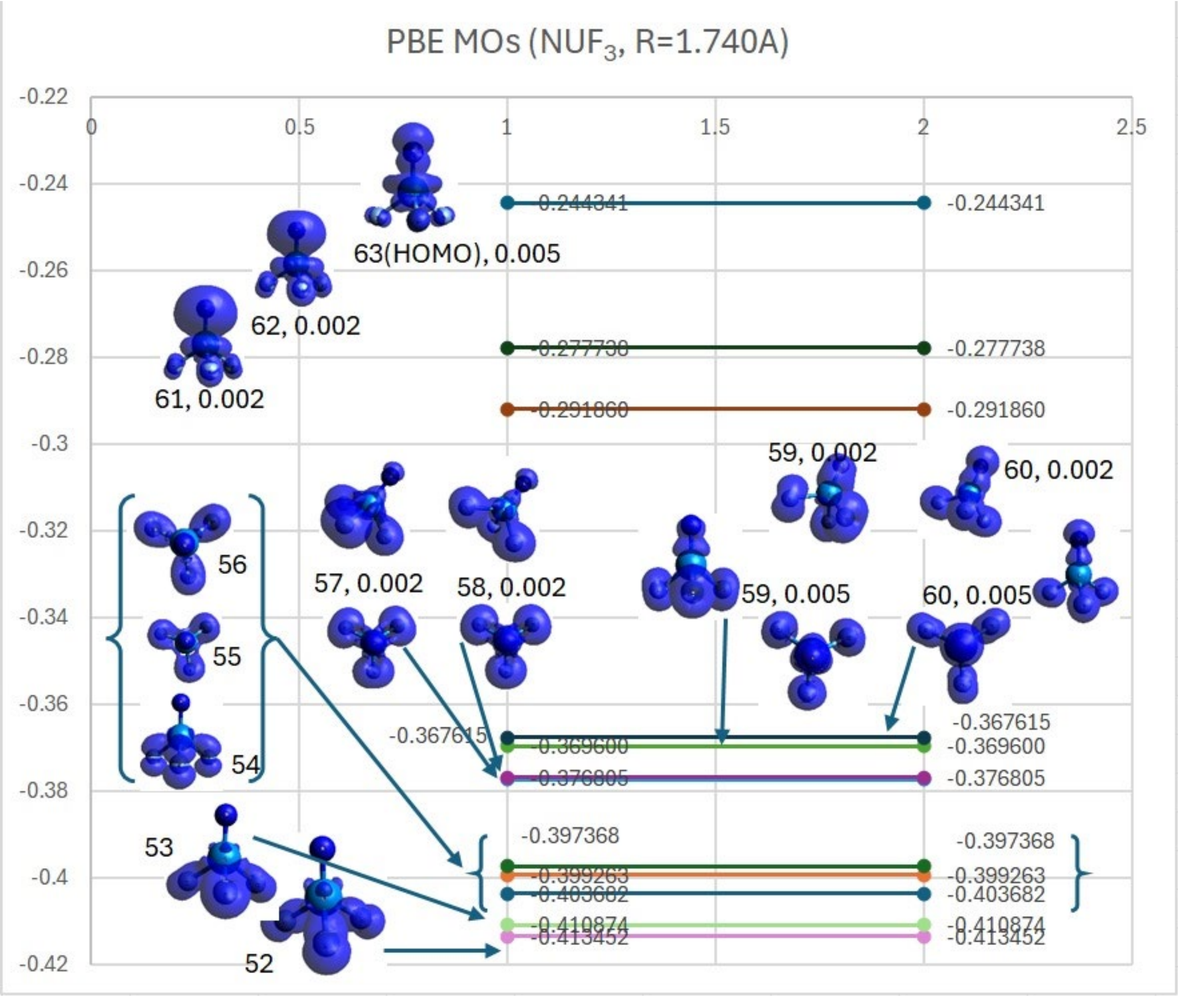

Fig. T14: NUF_3 orbital isodensities for Kramer pairs 52 to 63.

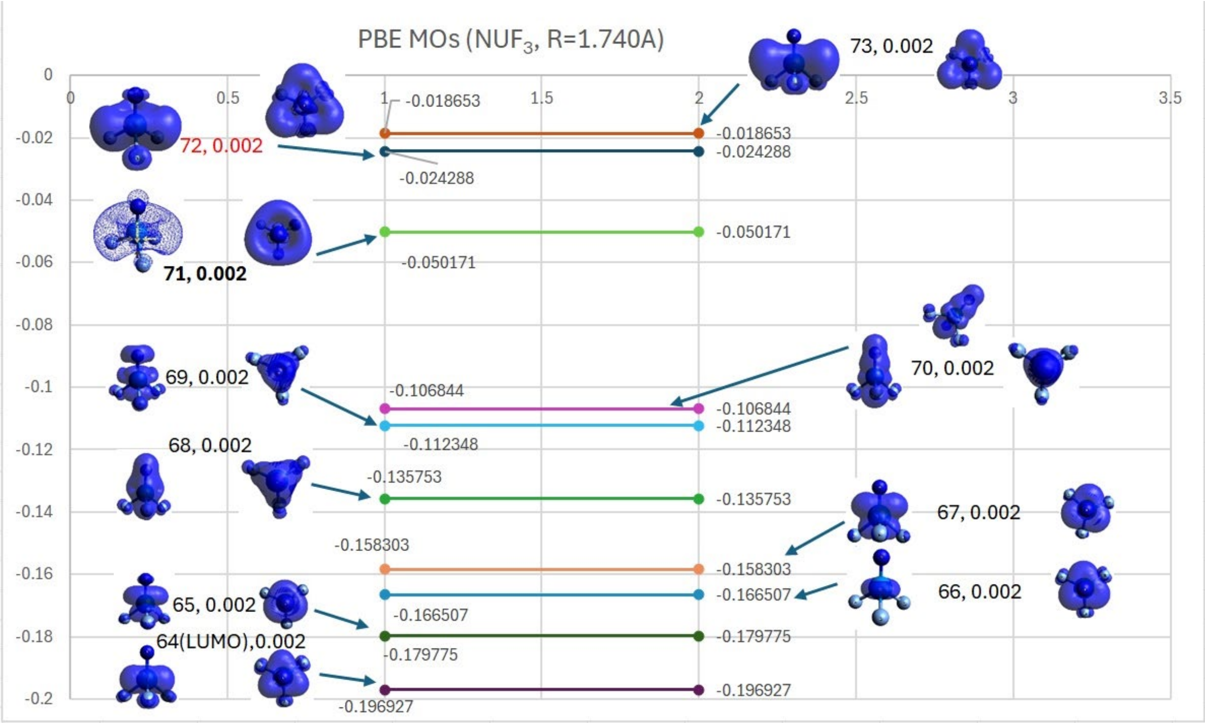

Fig. T15: NUF_3 orbital isodensities for Kramer pairs 64 to 73.

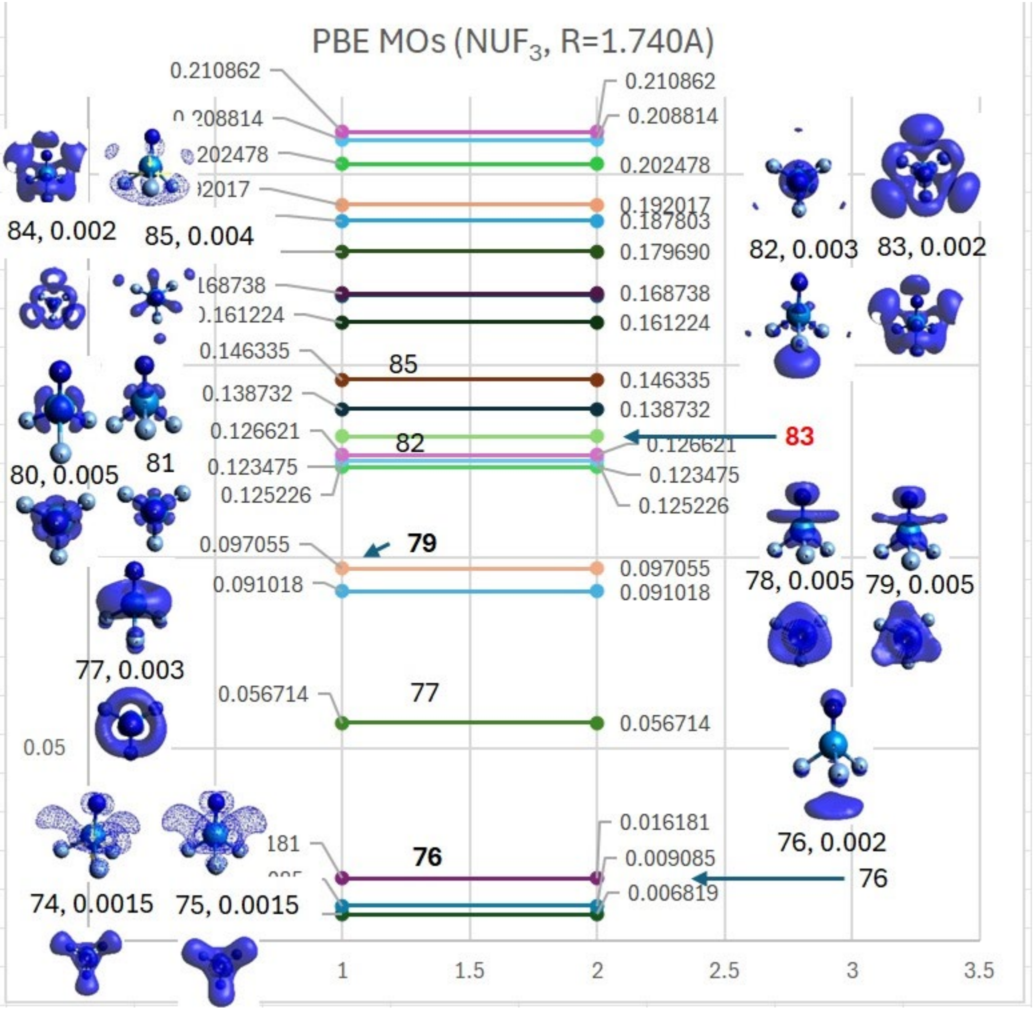

Fig. T16: NUF_3 orbital isodensities for Kramer pairs 76 to 85.

NUHFI molecule

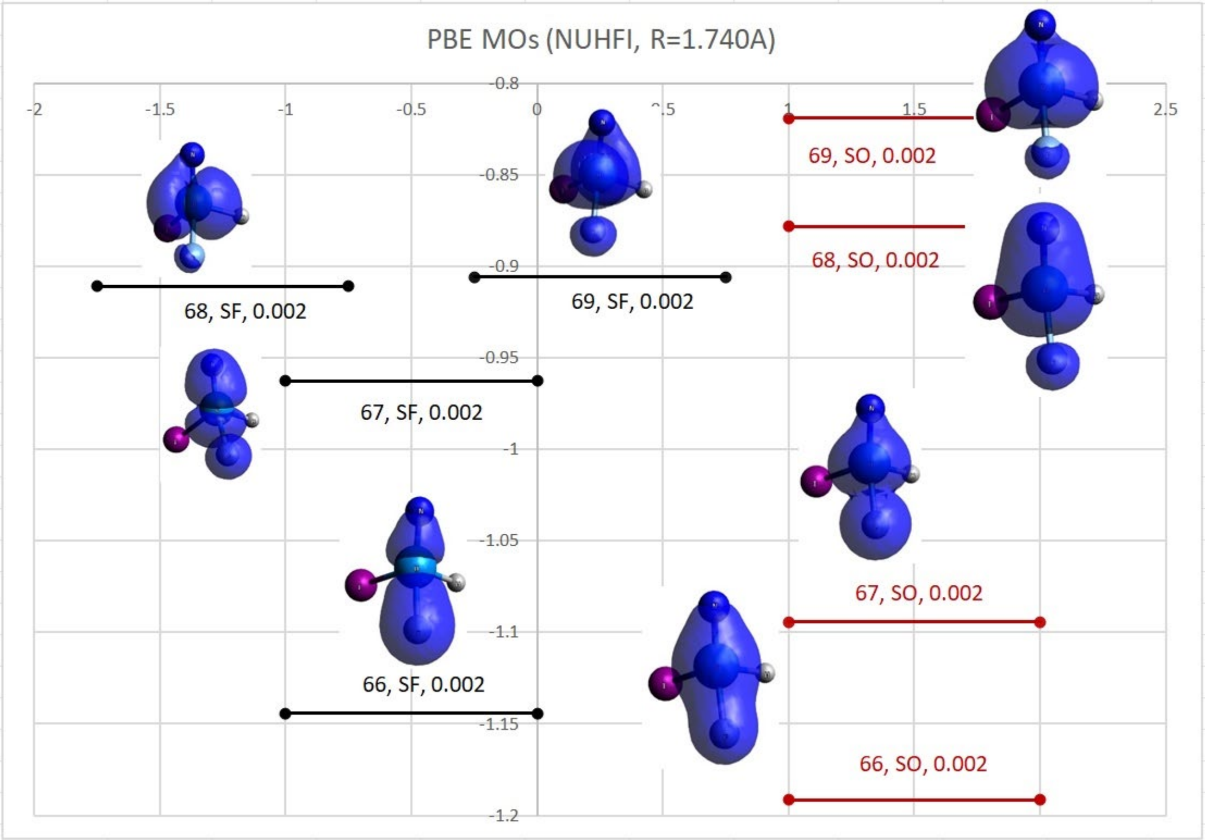

Fig. T17: NUHFI orbital isodensities (SF = spin-free case, SO = full 4c-Dirac-Coulomb Hamiltonian) for Kramer pairs 66 to 69.

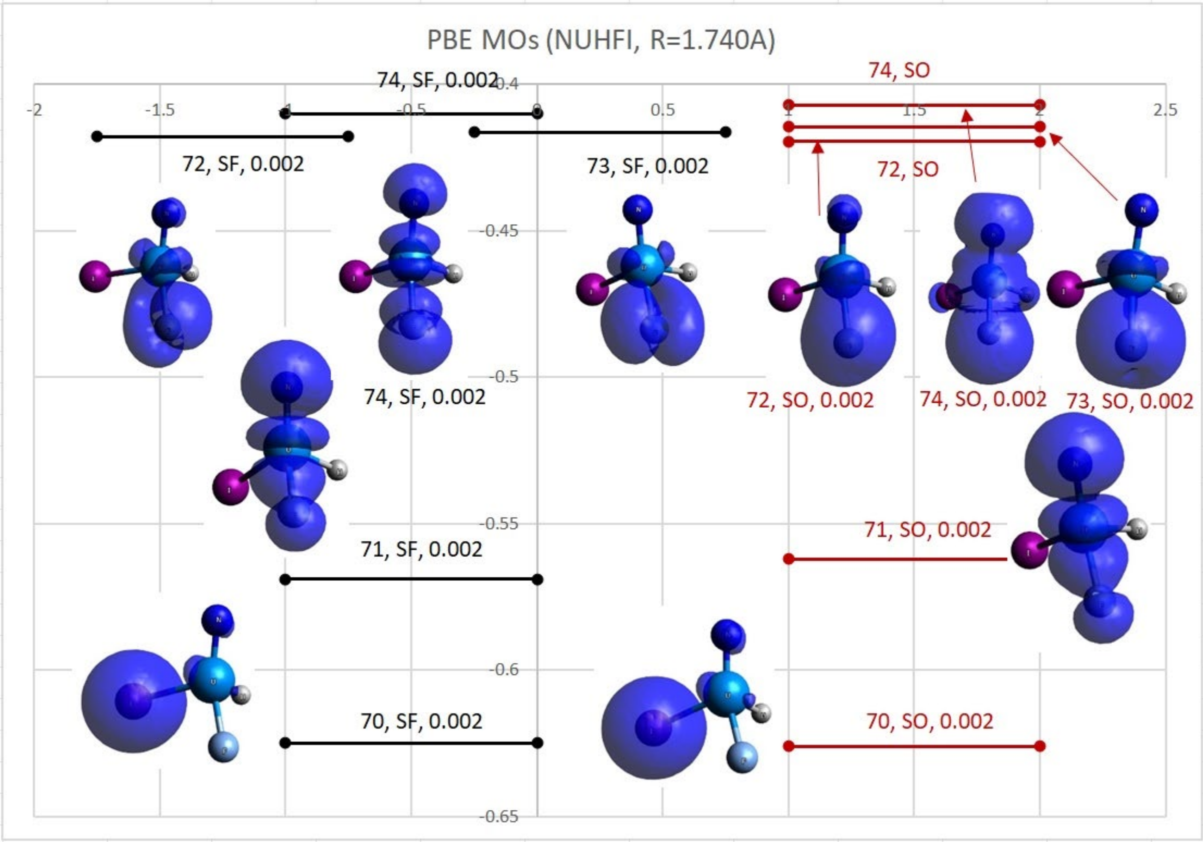

Fig. T18: NUHFI orbital isodensities for Kramer pairs 70 to 74.

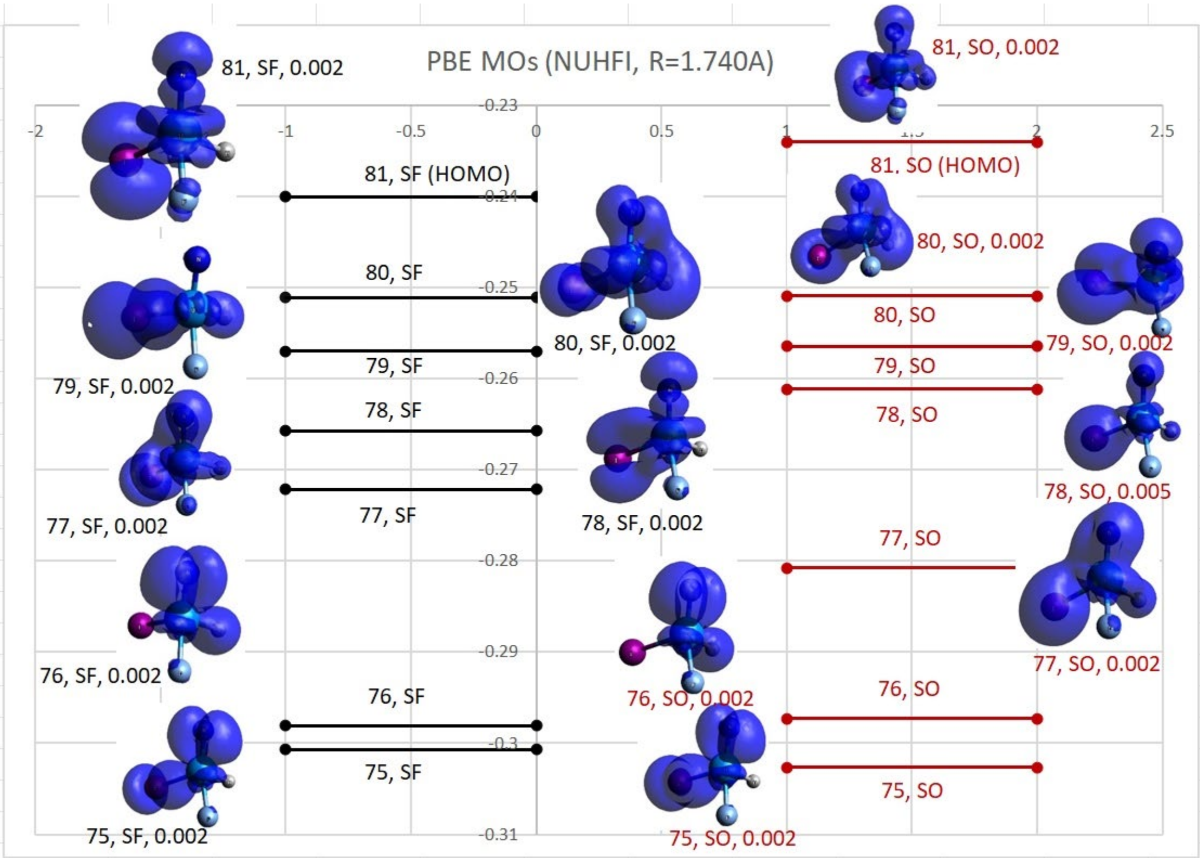

Fig. T19: NUHFI orbital isodensities for Kramer pairs 75 to 81.

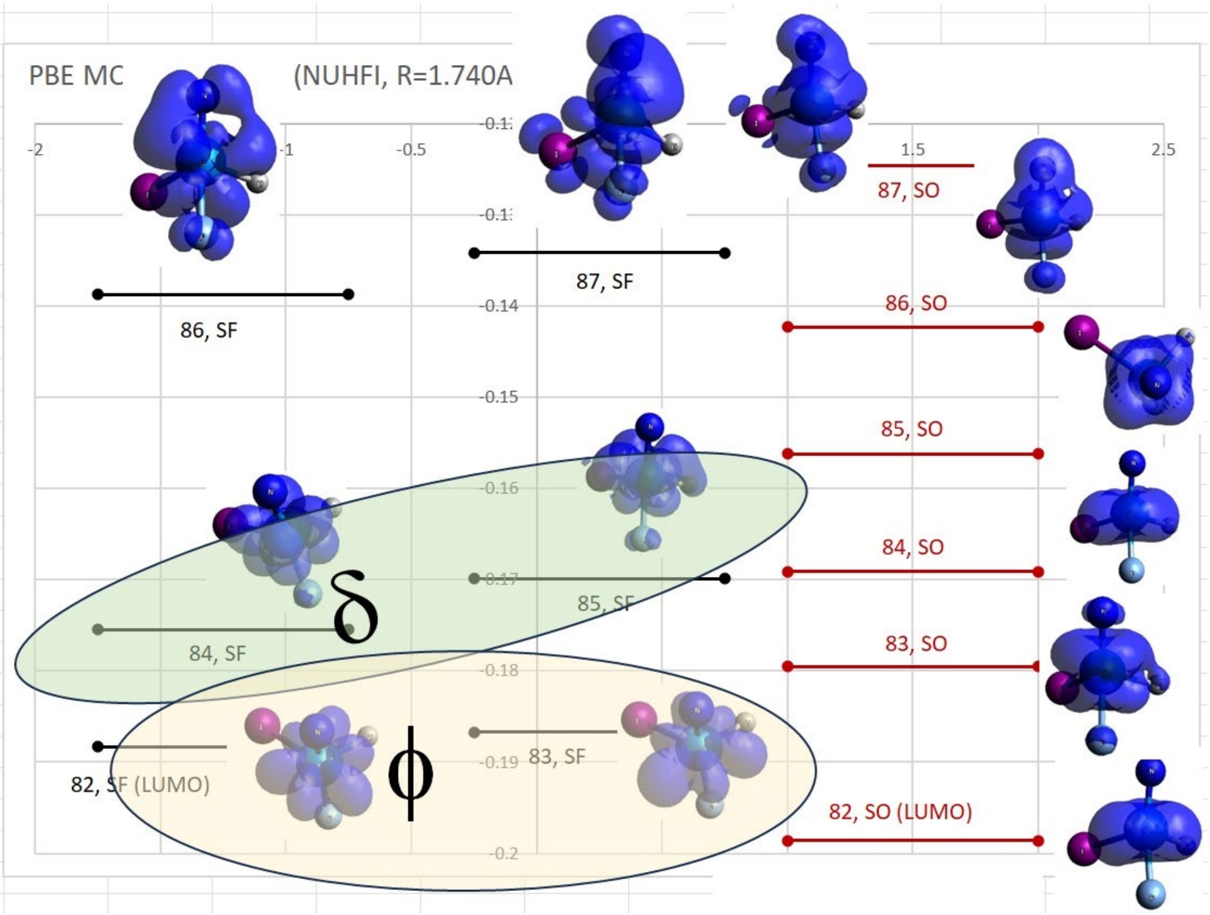

Fig. T20: NUHFI orbital isodensities for Kramer pairs 82 to 87.

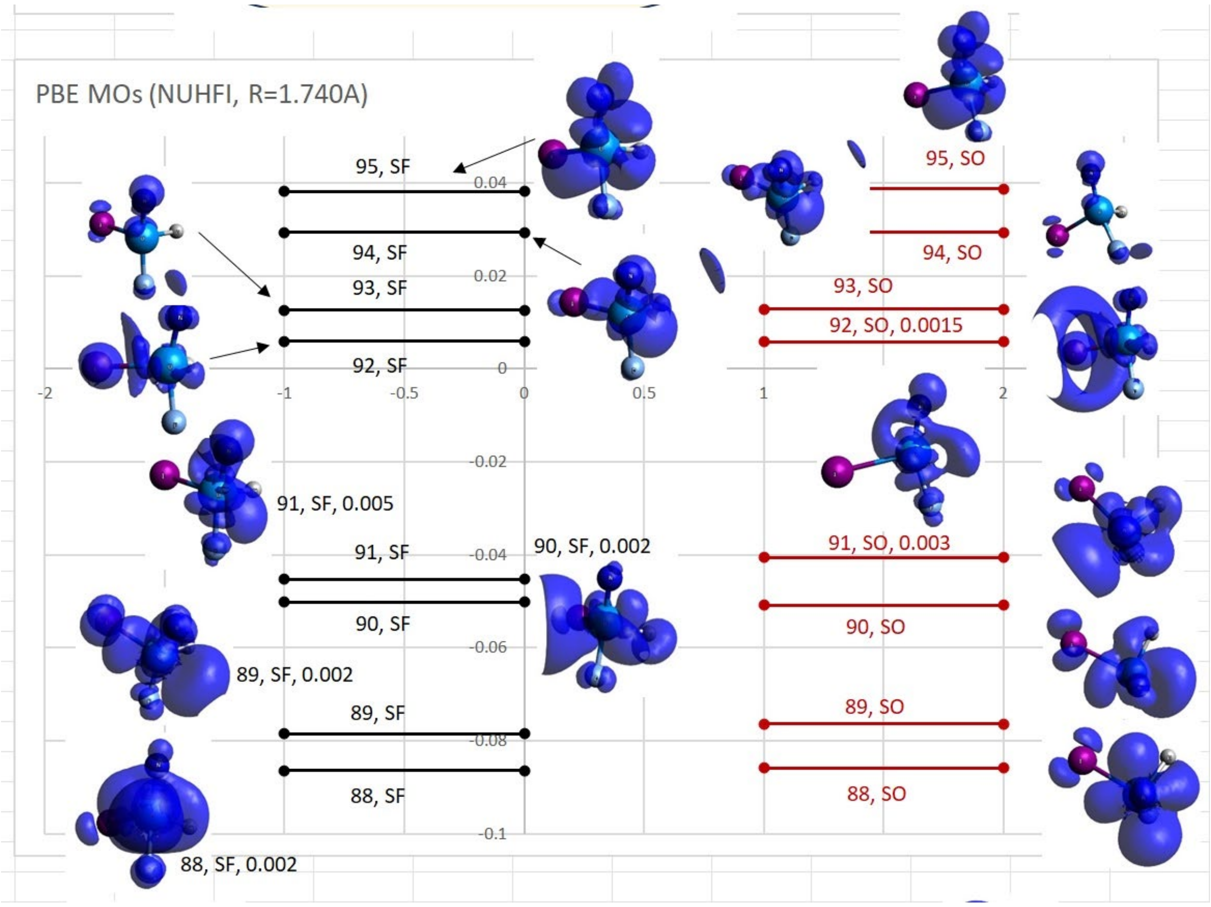

Fig. T21: NUHFI orbital isodensities for Kramer pairs 88 to 95.

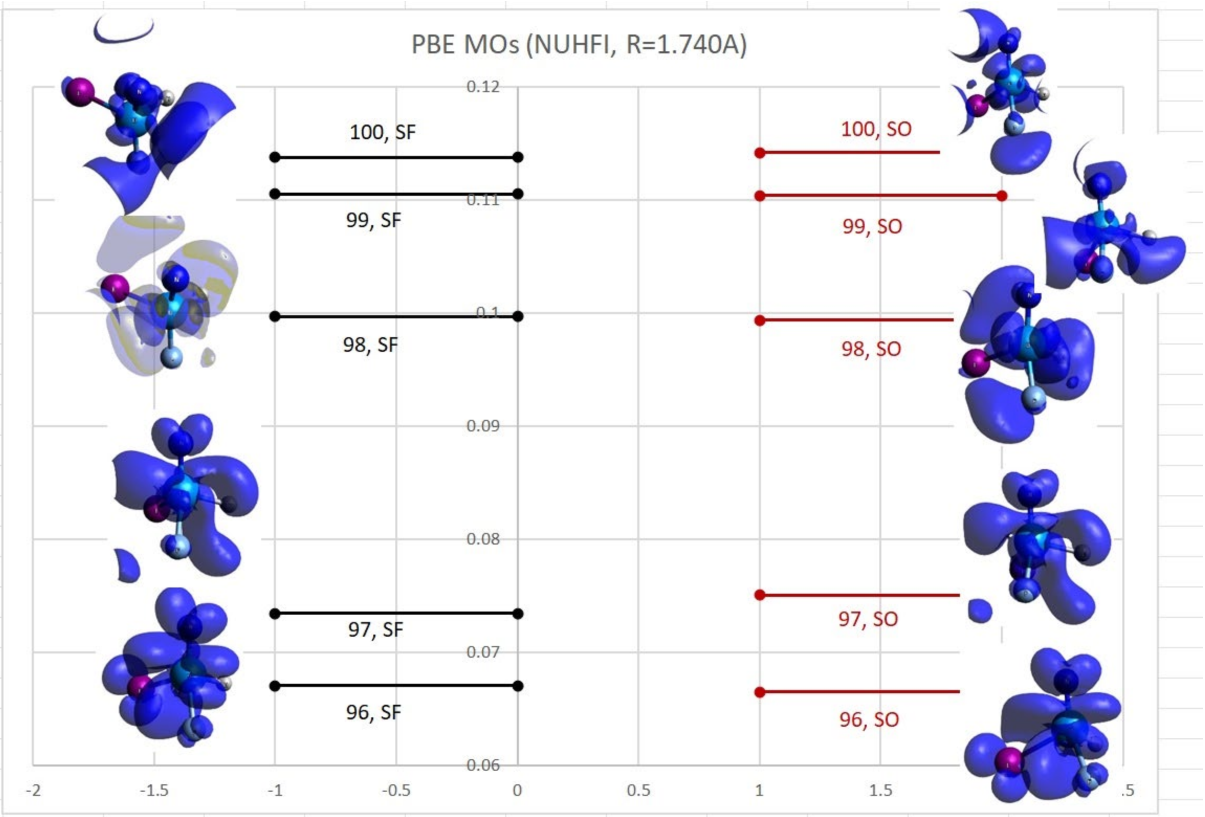

Fig. T22: NUHFI orbital isodensities for Kramer pairs 96 to 100.

The following figures show only the „SO“ case (corresponding to the article), the value after the Kramer pair index is the isodensity of the plot.

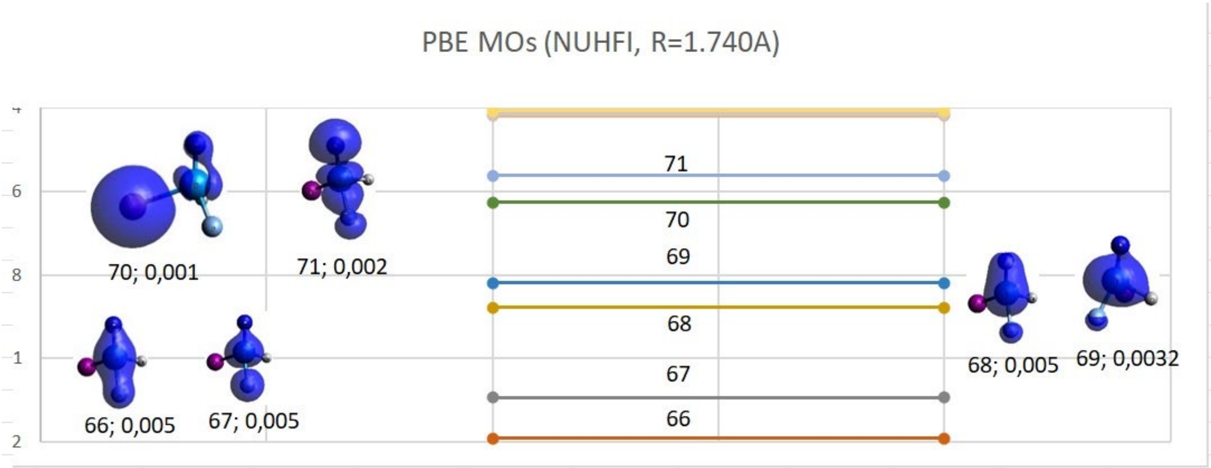

Fig. T23: NUHFI orbital isodensities for Kramer pairs 66 to 71 (only SO case).

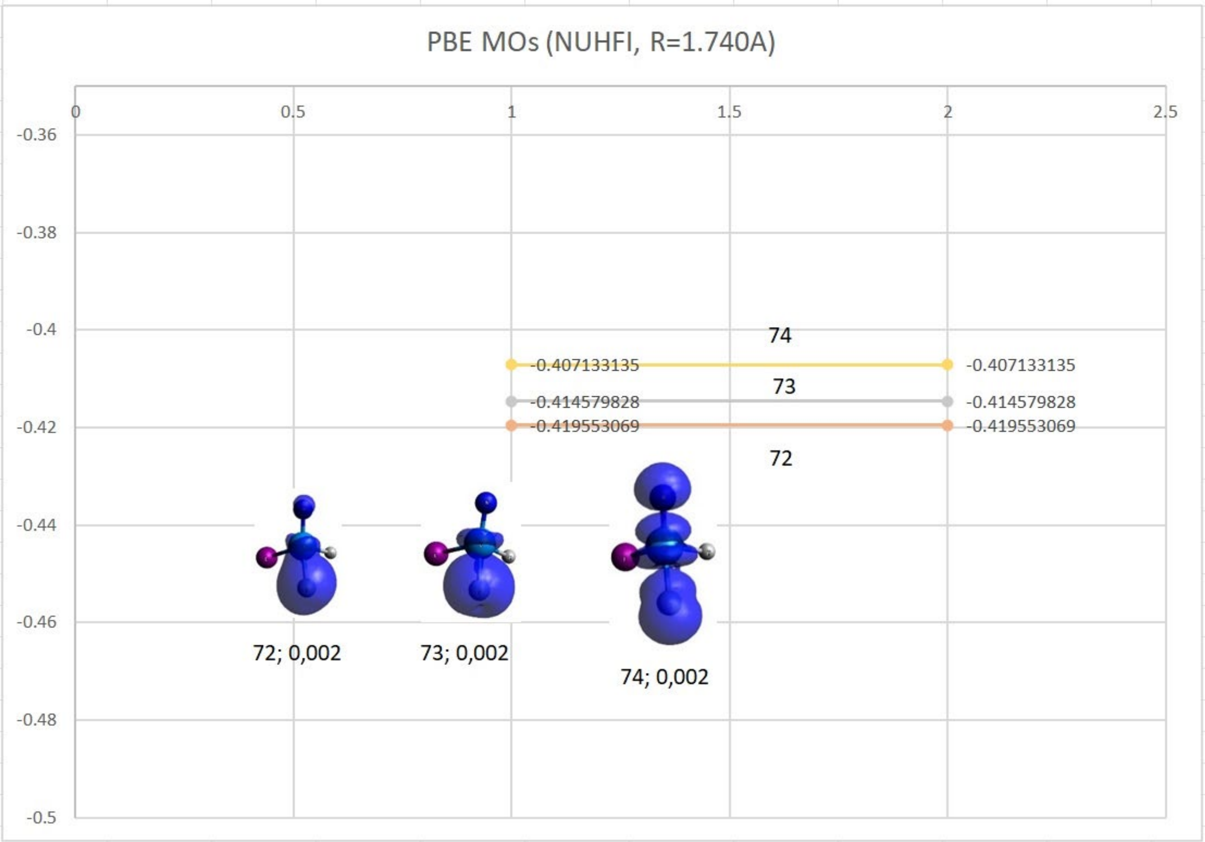

Fig. T24: NUHFI orbital isodensities for Kramer pairs 71 to 74.

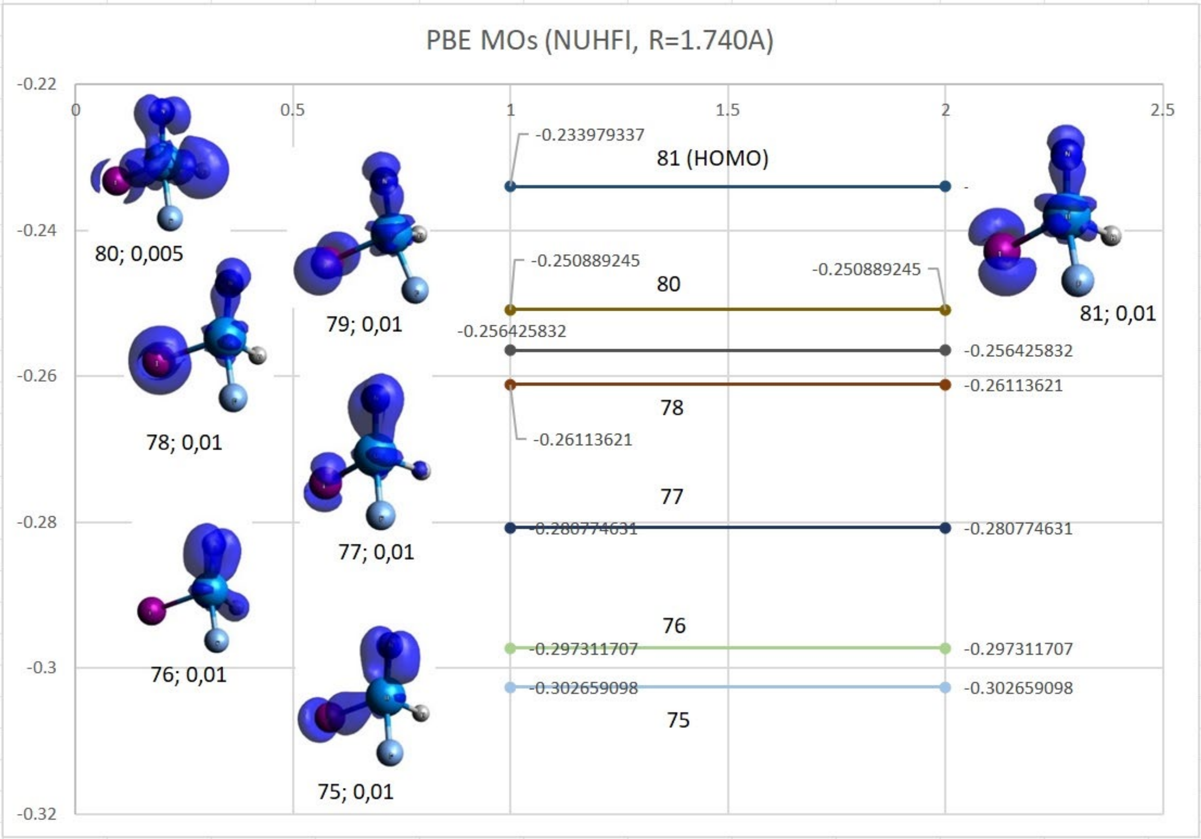

Fig. T25: NUHFI orbital isodensities for Kramer pairs 75 to 81.

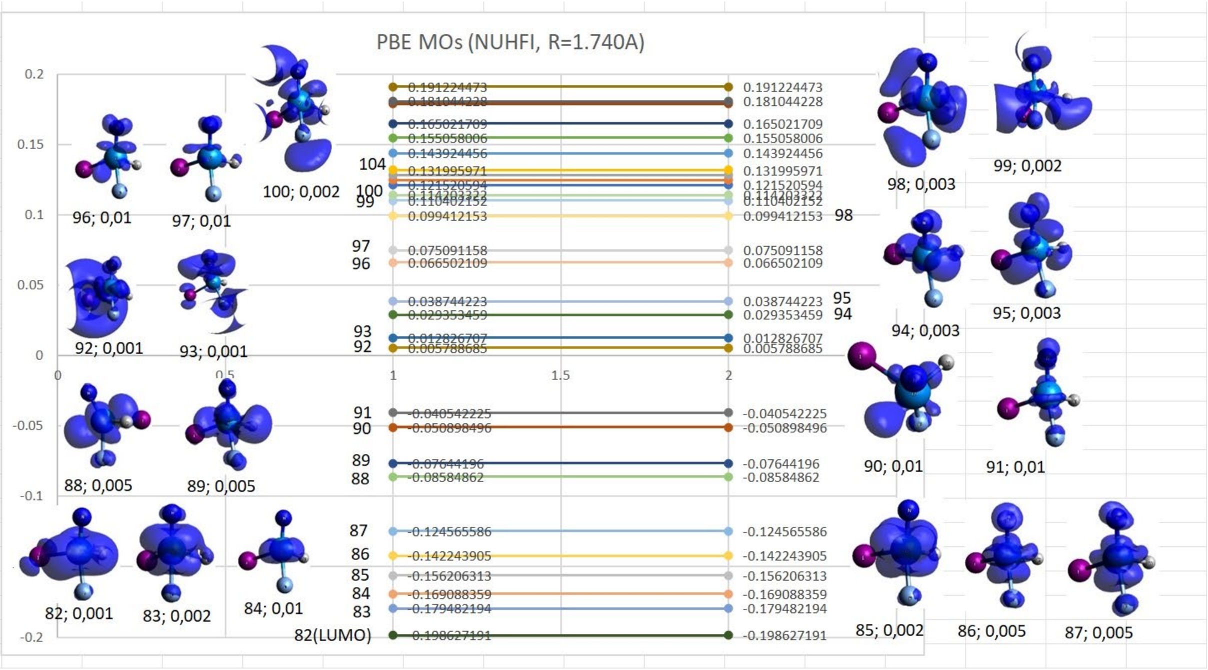

Fig. T26: NUHFI orbital isodensities for Kramer pairs 82 to 104.

TCC active space optimisation

SCF MO basis set (NUHFI)

TCCSD energies for different active spaces of frontier SCF molecular bispinors have been computed (plotted in the Fig. S4 below) and the minimal combination found so far corresponds to (28,39). In principle, the minimal combination could correspond to even greater active space (Fig. S4), but we could not afford to determine TCCSD energies for larger active spaces.

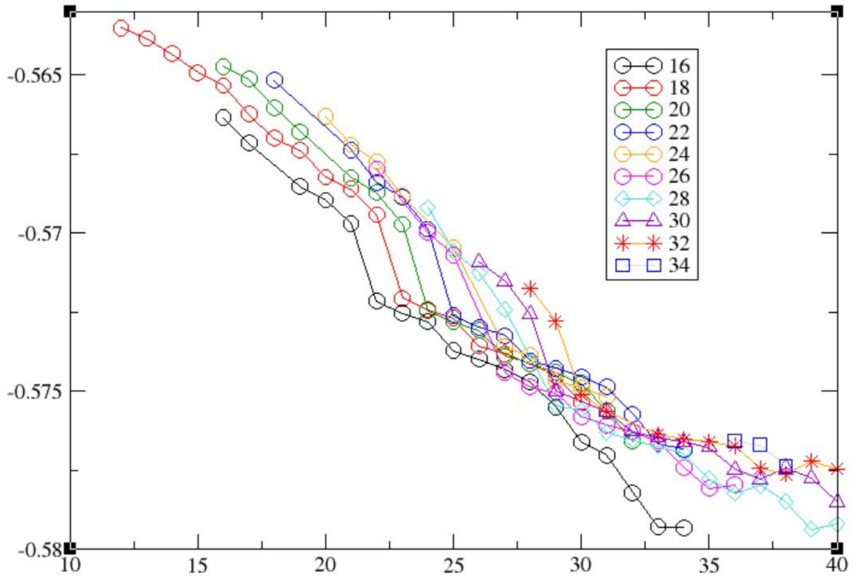

Fig. S4: Active space optimisation for (n,m)DMRG -TCCSD single point computation of NUHFI molecule (R = 1.700 A) in SCF/DZ basis set (MO/AO), bond dimension M = 1024, CC orbital space 47..163. Horizontal axis: m, legend: n, vertical axis: (n,m)DMRG -TCCSD(47..163) + 35330 hartree offset.

MP2NO basis set (NUHFI)

TCCSD energies for different active spaces of frontier MP2NO molecular bispinors have been computed (plotted in the Fig. S5 below) and the minimal combination corresponds to (28,20).

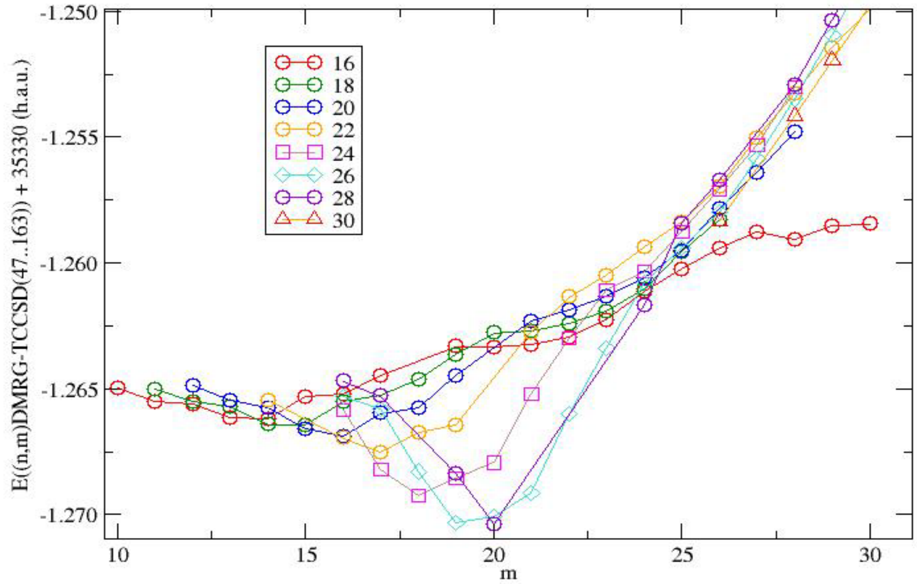

Fig. S5: Active space optimisation for DMRG(n,m)-TCCSD single point computation of NUHFI molecule (R = 1.700 A) in SCF/DZ basis set (MO/AO), bond dimension M = 1024, CC orbital space 47..163.

Single orbital entropy (NUHFI, PBE-KS bispinors)

The single orbital entropy profile for r = 1.4 A presented in the main article in Fig. 3 (for various bond dimensions up to M = 256 – which is similar as for M = 128) is supplemented here by single orbital entropy profiles for near-equilibrium distance of r = 1.7 A and dissociation region point of r = 4.8 A. The selection of active space shall be based on the combination of entropy profiles across all studied molecular geometries.

Single orbital entropy (NUHFI, PBE-KS bispinors)

The single orbital entropy profile for r = 1.4 A presented in the main article in Fig. 3 (for various bond dimensions up to M = 256 – which is similar as for M = 128) is supplemented here by single orbital entropy profiles for near-equilibrium distance of r = 1.7 A and dissociation region point of r = 4.8 A. The selection of active space shall be based on the combination of entropy profiles across all studied molecular geometries.

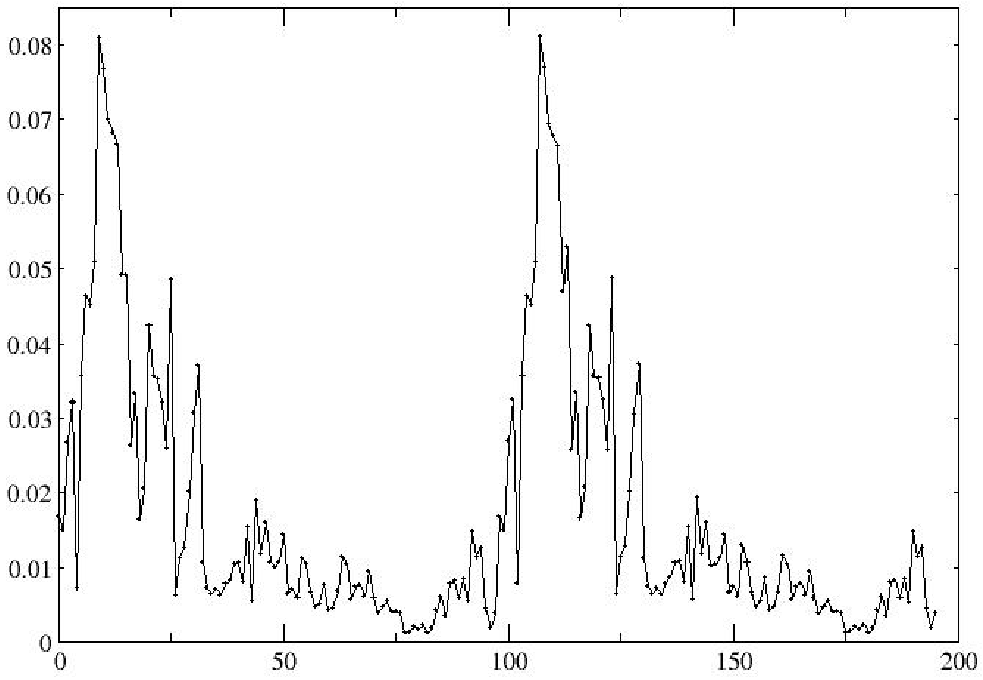

Fig. S6: DMRG(32,98) (single orbital) entropy profile at r_{N-U} = 1.7 Å for bond dimension M = 128.

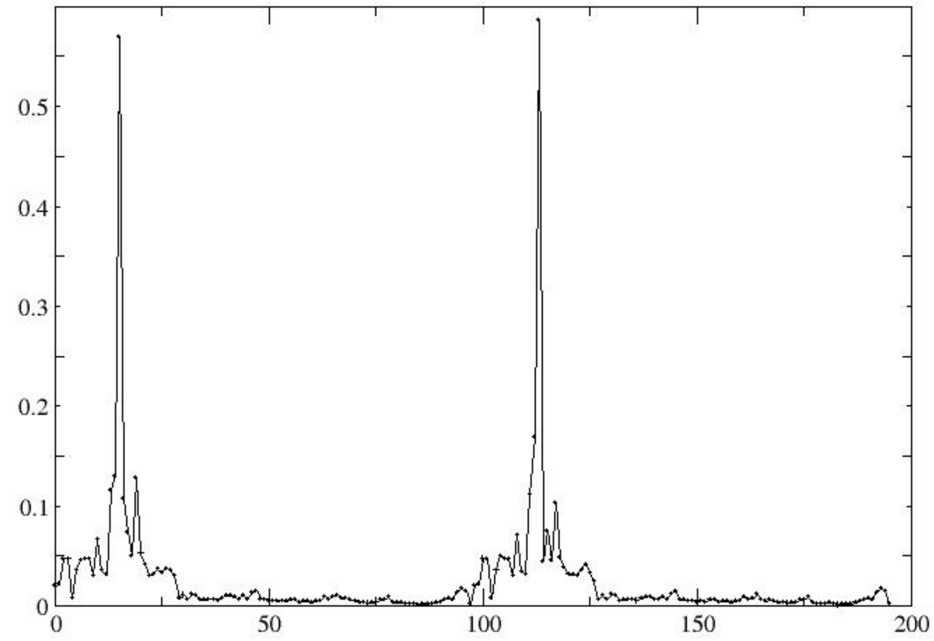

Fig. S7: DMRG(32,98) (single orbital) entropy profile at r_{N-U} = 4.8 Å for bond dimension M = 128.

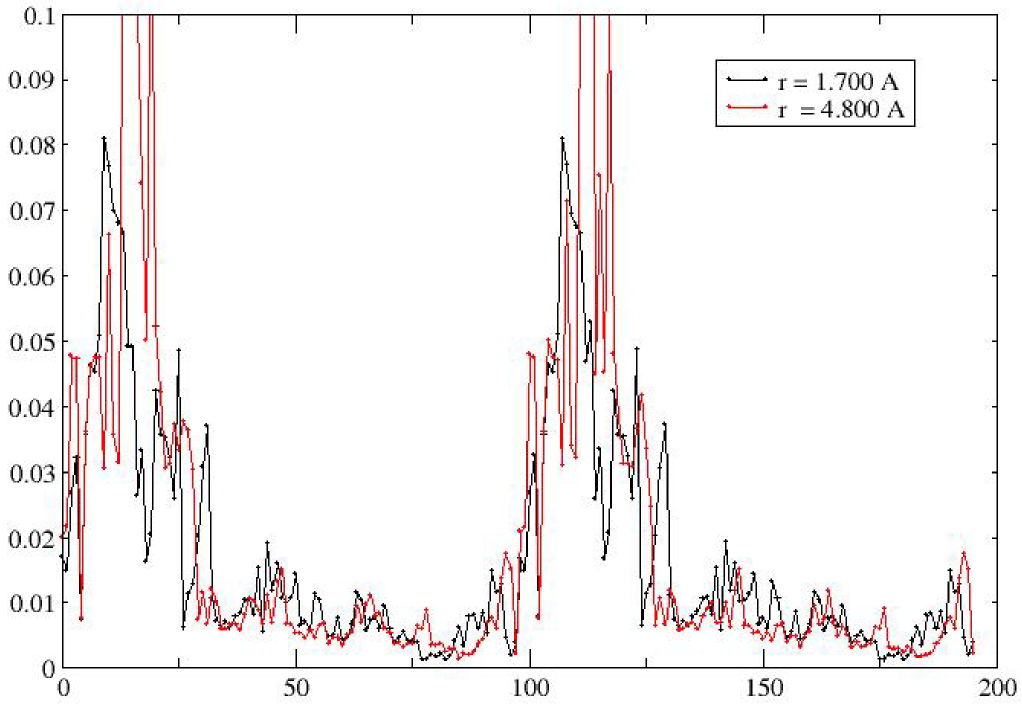

Fig. S8: DMRG(32,98) (single orbital) entropy profiles at r_{N-U} = 1.7 and 4.8 Å for bond dimension M = 128. The entropy axis cut at the same level as in Fig. 3 in the main article

From this comparison (Fig. S8, Fig. 3 in the main article), we can conclude that peak positions in entropy profiles do shift to higher orbital indices with increased bond length – in accordance with the greater contribution of virtual/antibonding orbitals in the dissociation region.

[Atkinson2018] B. E. Atkinson, H.-S. Hu, and N. Kaltsoyannis: Post Hartree–Fock calculations of pnictogen–uranium bonding in EUF3 (E = N–Bi), Chem. Commun. 54, 11100 (2018).

[CaoDolg2004] Xiaoyan Cao, Michael Dolg, J. Molec. Struct. 673 (2004) 203 - 209.

[Eichkorn1997] K. Eichkorn, F.Weigend, O. Treutler and R.Ahlrichs: Auxiliary basis sets for main row atoms and transition metals and their use to approximate Coulomb potentials.; Theor. Chem. Acc., 97, 119(1997).

[Hellweg2007] A. Hellweg, C. Hättig, S. Höfener and W. Klopper: Optimized accurate auxiliary basis sets for RI-MP2 and RI-CC2 calculations for the atoms Rb to Rn.; Theor. Chem. Acc., 117, 587(2007).

[Weigend1998] F. Weigend, M.Häser, H.Patzelt and R.Ahlrichs: RI-MP2: Optimized Auxiliary Basis Sets and Demonstration of Efficiency.; Chem.Phys. Lett., 294, 143 (1998).

[Weigend2002] F. Weigend: A fully direct RI-HF algorithm: Implementation, optimised auxiliary basis sets, demonstration of accuracy and efficiency.; Phys.Chem.Chem.Phys.,4, 4285(2002).

[Weigend2005a] F. Weigend, R. Ahlrichs: Balanced basis sets of split valence, triple zeta valence and quadruple zeta valence quality for H to Rn: Design and assessment of accuracyPhys. Chem. Chem. Phys. 7, 3297 (2005), DOI: 10.1039/b508541a.

[Weigend2006] F. Weigend: Accurate Coulomb-fitting basis sets for H to Rn.; Phys. Chem. Chem. Phys., 8, 1057(2006).

[Weigend2008] F. Weigend: Hartree–Fock Exchange Fitting Basis Sets for H to Rn.; J. Comput. Chem., 29, 167(2008).
